# Ceramic Microfiltration Membranes in Wastewater Treatment: Filtration Behavior, Fouling and Prevention

**DOI:** 10.3390/membranes10090248

**Published:** 2020-09-22

**Authors:** Mohammed Wali Hakami, Abdullah Alkhudhiri, Sirhan Al-Batty, Myrto-Panagiota Zacharof, Jon Maddy, Nidal Hilal

**Affiliations:** 1 Chemical Engineering Technology Department, Jubail Industrial College, Jubail Industrial City 31961, Saudi Arabia; hakami_mw@jic.edu.sa (M.W.H.); batty_sa@jic.edu.sa (S.A.-B.); 2 King Abdulaziz City for Science and Technology (KACST), National Center for Desalination & Water Treatment Technology, Riyadh 12354, Saudi Arabia; khudhiri@kacst.edu.sa; 3 Sustainable Environment Research Centre (SERC), Faculty of Engineering, Computing and Science, University of South Wales, Pontypridd CF37 1DL, UK; jon.maddy@southwales.ac.uk; 4 NYUAD Water Research Center, New York University, Abu Dhabi 129188, UAE; nidal.hilal@nyu.edu

**Keywords:** ceramic membranes, filtration, microfiltration, cleaning, tubular, pores, flux

## Abstract

Nowadays, integrated microfiltration (MF) membrane systems treatment is becoming widely popular due to its feasibility, process reliability, commercial availability, modularity, relative insensitivity in case of wastewater of various industrial sources as well as raw water treatment and lower operating costs. The well thought out, designed and implemented use of membranes can decrease capital cost, reduce chemical usage, and require little maintenance. Due to their resistance to extreme operating conditions and cleaning protocols, ceramic MF membranes are gradually becoming more employed in the drinking water and wastewater treatment industries when compared with organic and polymeric membranes. Regardless of their many advantages, during continuous operation these membranes are susceptible to a fouling process that can be detrimental for successful and continuous plant operations. Chemical and microbial agents including suspended particles, organic matter particulates, microorganisms and heavy metals mainly contribute to fouling, a complex multifactorial phenomenon. Several strategies, such as chemical cleaning protocols, turbulence promoters and backwashing with air or liquids are currently used in the industry, mainly focusing around early prevention and treatment, so that the separation efficiency of MF membranes will not decrease over time. Other strategies include combining coagulation with either inorganic or organic coagulants, with membrane treatment which can potentially enhance pollutants retention and reduce membrane fouling.

## 1. Introduction

The continuously intensifying human population growth has led to the demand for clean water to be steeply increased while the gap between water demand and supply is getting wider [1,2]. In addition to conventional sources, other sources of water are currently being considered for use such as groundwater, water hold by dams, brackish water and water reuse generated by wastewater treatment. Given that underground water can be efficiently pumped to the surface even in remote areas using electrical or diesel generators driving various types of pumps [3], it can be a major auxiliary water source.

Wastewater and water treatment methods include both physical methods such as sedimentation and filtration (membranes, media filtration) and chemical methods such as coagulation, pH adjustments, addition of anti-scalants and acids [4,5]. Regardless of its domestic, municipal, or industrial origin, wastewater is a serious environmental constraint that dictates effective treatment for its safe discharge in the aquatic environment. Wastewater has been regularly identified among other substances as containing hazardous chemicals including metals (e.g., As, Pb, Cr, Cd and Zn), toxic compounds such as endocrine disruptors, dyes and a strong, pungent odor due to the high content of organic matter. On the other hand underground water or also known as ground water is, though, prone to contaminants either naturally occurring or augmented by human activity to pollutants and hazardous substances that need to be removed as for the water to be constituted safe for consumption. Heavy metals, such as mercury, copper and lead can cause serious health problems in excessive amounts, including reduced growth and development, autoimmune diseases, cancer, organ damage, nervous system damage and in extreme cases, death [6]. Another main contaminant is natural organic matter (NOM), synthetic detergents, nutrients such as phosphate and ammonia, heavy metals (HM), coliform bacteria as well as other microorganisms. NOM is a complex mixture of compounds including fulvic acid, humic acid (HA) [7] and humin formed through decay of plant and animal material in nature and is present in numerous sources. It is composed of a range of small, low molecular weight species such as carboxylic amino acids and proteins and larger, higher molecular weight species (from 0.5–30 kDa) such as humic and fulvic acids in high concentrations [8]. 

Currently, integrated membrane systems treatment is becoming widely popular due to their feasibility, process reliability, commercial availability, modularity, relative insensitivity in case of raw water processing and lower operating costs. Integrated membrane systems have also been proposed as the most suitable solution for decentralized wastewater treatment [9,10], should this be needed due to generation of waste from rural industry (farming, livestock breeding, biogas generation through anaerobic digestion), other industrial waste producing activities such as food, beverage and dairy processing or the local population growth and activities (correctional facilities, health and wellbeing settlements, community centers) [10]. The well thought out, designed and implemented use of membranes can decrease capital cost, reduce chemical usage, and require little maintenance [10].

Membranes can offer high productivity both in terms of product recovery as well as pollutants retention and low operational cost compared to other competing technologies, since there is no water phase change and often minimal or no use of chemical additives [11]. Among the numerous material arrangements for membranes, ceramic membranes are more and more employed in the drinking water and wastewater treatment industries when compared with organic and polymeric counterparts due to their resistance to extreme operating conditions and numerous available and sustainable cleaning protocols [12]. This allows longer service lifetime and highly efficient filtration performance. Tubular membranes modules provide a modest surface area to volume ratio, and thus the highest cost per unit area of all cylindrical membrane geometries, but also provide potentially the greatest turbulence promotion and the best access to the membrane surface [13].

Regardless of their many advantages, membranes are susceptible to fouling, an action that can be detrimental for the successful and continuous plant operations [14]. Several strategies such as chemical cleaning protocols and backflushing with air or liquids, can be put in place so the separation and mechanical characteristics of membranes should not change in the long run. Other strategies include combining coagulation with either inorganic or organic coagulants, with membrane treatment which can potentially enhance pollutants retention and reduce membrane fouling. Precipitation of coagulated colloids at high coagulants concentration or high ionic strength in the feed reduces the feasibility of inorganic substances as coagulation aids, but organic coagulants have recently been preferred as they do not experience high precipitation, which gives them an easier to handle constitution [15].

Therefore, in this narrative review the authors will attempt to explain the phenomenon of ceramic microfiltration membranes fouling occurring mainly in wastewater and water treatment. The review will be examining the pressing matter of water scarcity across the world, setting the tone of the necessity of usage of alternative source of water, introducing the concept of pressure driven membrane filtration, its types and categories, discussing ceramic microfiltration membranes and the occurring fouling phenomenon as well as current applied in practical methods of its treatment, attempting to extrapolate greater awareness in a relatively under-investigated matter within the last decade (as well as collating the main prevention and management methods, such as coagulation and cleaning. 

## 2. Water Crisis

Climate change has not only brought great attention in greenhouse (GHG) emissions and their detrimental environmental impact but also to the necessity to maintain clean water supply for drinking purposes as well as for irrigation, agriculture, and industrial uses. For adequate living standards, countries need to maintain annual renewable water resources (ARWR) of at least 2000 m^3^/capita, while a country with ARWR of 1000–2000 m^3^/capita can possibly suffer occasional and localized water shortages [16].

With 1000 m^3^/capita being the threshold critical value [17,18,19,20] countries with less than this will suffer serious water shortages that would strongly impend economic development, human health, and well-being. With an ARWR less than 500 m^3^/capita, a country is likely to experience ‘absolute water scarcity’.

In the EU, changing weather conditions, droughts and water shortages have shown a substantial increase over the past fifteen years, with increasing severity clearly demanding judicious, efficient and effective water management. Within the Mediterranean basin over 50% of the population is evidently affected by water stress during the summer months, while the phenomenon is no longer confined to certain areas but will be most possibly affecting at least half Europe’s river basins by 2030 [21]. It has been found that a low stream flow conditions can lead to an imposed wastewater reuse rates in drinking water treatment plants of up to 20% [22]. This demonstrates why the effective treatment of wastewater is such a necessity.

Water reuse is supported by a fit-for-purpose approach, based on risk assessment, therefore achieving risk minimization through multi-barrier criteria, including water-treatment barriers and physical barriers to limit contact. Until recently, the focus on water reuse concerns has been on the well documented risks of microbiological parameters to human health [23,24]. Other parameters are currently being investigated such as effluent organic matter (EfOM) control within the scope of reduction and removal in the treated water brings significant benefits, namely by decreasing color, odor, and synthetic organic compounds [25,26]. Conventional methods of disinfection, such as chlorination, produce carcinogenic and hazardous by-products such as trihalomethane (THM), haloacetic acids (HAA), haloacetonitriles and haloketones which have an adverse effect on human health [27,28,29,30]. EU countries, including the United Kingdom, have regulated the levels of THM in drinking water at 100 μg/L, while in the US, the US Environmental Protection Agency (USEPA) has set the levels at 80 μg/L with the HAA limit is 60 μg/L [31,32]. Membrane filtration has been successfully used in water treatment for NOM removal [31,32,33], with microfiltration (MF) being one of the most efficient membrane processes, although fouling due to NOM has been identified as a major problem as NOM particles tend to bind not only among each other or with other substances but also on the membrane surfaces [34,35,36].

Water reuse is a vital tool for extending the water life cycle and in full compliance with the circular economy incentives but it has not been adapted to its full potential. Climate change has driven new global strategies, for instance those of the International Organization for Standardization (ISO/TC 282 Water Reuse) and, at an EU level, the targeting of a substantial increase in recycling and safe reuse globally by 2030 (United Nations (UN) Sustainable Development Goal on Water, SDG 6). These led to the establishment of water reuse as a top priority area (Strategic Implementation Plan of the European Innovation Partnership on Water) and the specific objective of water reuse maximization (Blueprint to safeguard Europe’s water resources). Therefore, the European Commission proposed in May 2018 new rules to stimulate and facilitate water reuse in the EU for agricultural irrigation [37,38,39,40].

Other than the European South , climate change is also strongly affecting the already challenged in terms of water scarcity countries of the MENA (Middle East/North Africa) region (i.e., Algeria, Bahrain, Djibouti, Egypt, Iran, Iraq, Israel, Jordan, Kuwait, Lebanon, Libya, Morocco, Oman, Palestinian territories of Gaza and West Bank, Qatar, Saudi Arabia, Syria, Tunisia, United Arab Emirates, Yemen). The MENA region has 6% of the existing global population, but only 1% of the world’s freshwater resources [41]. The countries in the region depend on seasonal rainfall, have very few rivers, some of which carry runoff from other countries, and often rely on fragile, occasionally non-renewable, aquifers. Currently MENA countries will have ARWR of less than 1000 m^3^/capita with a projected decline to below 500 m^3^ per capita by 2025. The increasing competition for good quality water among different water-use sectors in the MENA region countries has decreased freshwater allocation to agriculture [42]. With the increase in wastewater generation, its productive use in agriculture has increased, as farmers have no alternative sources of reliable irrigation water [43,44,45] as the water taken away from agriculture is then diverted to non-agricultural uses. Overall, information on the productivity potential of wastewater, and its impacts on the environment, social and economic conditions of the dependent farming communities, is limited [46]. 

## 3. Membrane Technology and Applications

Microfiltration (MF), ultrafiltration (UF), and nanofiltration (NF) membrane separation processes are strongly emerging technologies that can be used in several separation processes. Membrane processes are progressively being used further in several fields substituting conventional concentration, separation and purification techniques. Nowadays, membrane processes can be found in numerous industries including water treatment for domestic and industrial water supplies, waste treatment (separation of salt or other minerals, deionization), chemical (organic material separation, gas separation, recovery and recycle chemicals), pulp and paper (replacing the evaporation process, fiber and chemicals recovery), leather and textile (sensible heat recovery, pollution control and chemicals recovery), food and beverage, metallurgy (metal recovery, pollution control, air enriching for combustion), pharmaceutical, automotive, diary, food and biotechnological (separation, purification, sterilization and by-product recovery), medical (artificial organs, control release, pharmaceutical, blood fractionation, sterilization and water purification) and the petrochemical industries [47].

There are numerous advantages that benefit from the use of membrane technologies, including their easy combination with other processes (hybrid processing), continuous separation, easy up-scaling, separation under mild conditions, low energy consumption and nonrequirement of additives. The growth and application of membrane technology is also pushed by the demand on industry for improved environmental solutions and cleaner technology.

## 4. Filtration

Filtration is a physical process that involves the separation (removal) of particulate and colloidal matter from a liquid. Filters are categorized into three general groups [38,39,40]: (1) depth filtration (2) surface filtration and (3) membrane filtrations. Depth filtration includes the removal of suspended materials within and on the surface of the filter bed. Sand and anthracite are usually used as filter media. In surface filtration, the suspended material is eliminated by straining through an exterior surface (e.g., filter cloth, diatomaceous earth filtration) [48]. The range of particle sizes in membrane filtration is extended to include dissolved constituents (typically to 1.0 μm). Membranes serve as selective barriers that allow the passage of constituents and retain other constituents. MF, UF, NF and reverse osmosis (RO) are operated with a hydrostatic pressure difference as the most used membrane driving forces in water and wastewater treatment [38,39,40]. The general characteristics of the varying membrane processes [49,50] are further reported in Table 1. It is necessary to operate a MF process with high surface velocity and low TMP. However, an optimization for the forces is needed for appropriate operation. Tangential flow across the membrane surface is preferable since it provides a continuous scouring action and hence reduces the membrane fouling layer due to feed stream debris and macromolecules.

### 4.1. Pressure Driven Membrane Processes

Membranes have been defined as engineered barriers that remove colloids, molecules or salt [51,52] using a non-fibrous, engineered barrier, through a size exclusion mechanism. Based on pore size, shape and chemical/physical properties, membranes can separate different particles, organisms and chemical species. These systems of membrane filtration are commonly known as pressure or vacuum driven processes.

**Figure 1:**
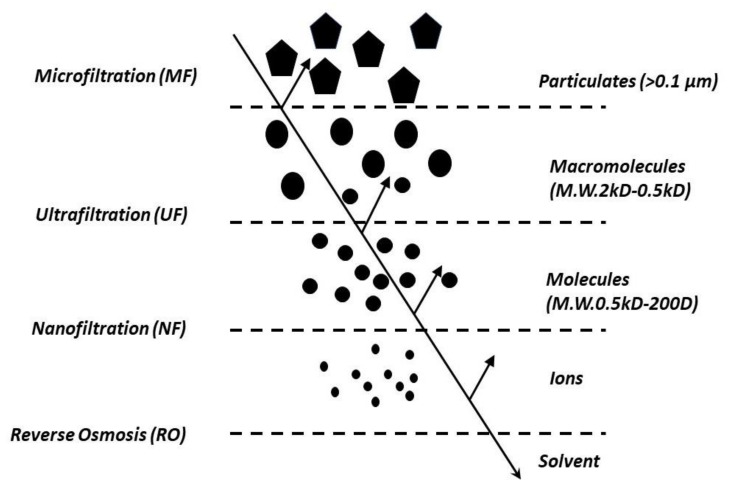
Schematic representation of microfiltration (MF), ultrafiltration (UF), nano filtration (NF) and reverse osmosis (RO) separation principles [58,59].

Membrane-driven processes (Figure 1) can conveniently remove various-sized organic matter, from small solutes (through NF) to macromolecules (through UF) or suspended matter (through MF) [53]. The permeate obtained after undergoing microfiltration and ultrafiltration can be feasibly reused in different stages, including rinsing, washing, and cleaning of industrial plants [54,55].

Applied pressure, forces the solvent and various solute molecules through the membrane, whereas other molecules are impermeable to various extents dependent on the structure of the membrane. Pore sizes are reduced further down the filtration ladder, thus the resistance of the membranes to mass transfer increases and so, the applied pressure has to be increased to obtain the same flux. All these processes are well-established technologies developed at all levels of industry.

Physical treatments such as sedimentation, flotation, and adsorption, as well as barriers such as bar racks, screens, deep bed filters, and membranes are the methods of choice for the purification of surface water and wastewater, due to their low costs and minimal environmental impacts. The application of membrane technologies to wastewater treatment has expanded over the last few decades, with continuous reduction of their costs and extension of the application possibilities [56]. Nominal or absolute pore sizes are often used when describing filtration capabilities of membrane materials. However, this number does not indicate the removal efficiency of the membrane. Filtering particles close in size to the pore distribution of the membrane can get quite complicated, since often particles that are smaller than most pores are removed not through sieving but through probabilistic interception in the depth of the filter media. In addition, particles may be excluded through electrostatic repulsion and adsorption to the membrane material. Over time, wear and tear takes its toll on the membrane through the deposition of particles and cake formation thus obscuring the pores of a membrane and increasing its removal efficiency [57].

**Table 1:** General characteristic of membrane processes [60,61,62,63,64,65].

Membrane Pores Size	MF	UF	NF	RO
Typical separation mechanism	Sieving	Sieving	Sieving, charge effect, adsorption, solution diffusion	Solution–Diffusion (diffusion limitation), convection)
Pore size (nm)	100–10,000	2–100	0.5–2	Unknown
Pressure (bar)	0.1–3	0.1–5	3–20	5–120
Permeability (l/h·m^2^·bar)	>1000	10–1000	15–30	0.05–1.5
Retention				
• Monovalent ions	–	–	–	+
• Multivalent ions	–	–/+	+	+
• Small organic compounds	–	–	–/+	+
• Macromolecules	–	+	+	+
• Particles	+	+	+	+
Energy consumption (kWh/m^3^)	0.4	3.0	5.3	10.2

### 4.2. Filtration Mode

There are usually two main types of filtrations carried out in membrane separation processes: dead-end and cross-flow filtration. The dead-end filtration is normally used on small scales in laboratories whereas cross-flow filtration is the main process used on large scales in many industries including desalination [66,67].

### 4.3. Dead-End Mode

Dead-end mode is a filtration method where the complete feed flow is forced perpendicular to the membrane surface, which allows the retained matter to build up on the membrane surface, due to clogging, and form a type of cake layer. The formed cake layer thickness increases with filtration time and consequently the permeate recovery rate decreases with an increased cake layer thickness. Dead-end mode is considered as the most basic form of filtration. It could be a practical technique for concentrating compounds [68]. 

### 4.4. Cross-Flow Mode

**Figure 2:**
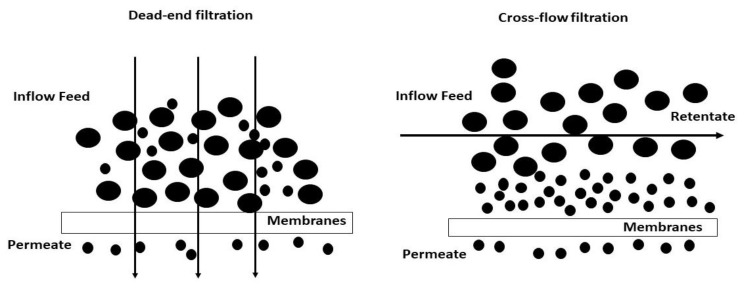
Mechanism of cross-flow filtration and dead-end filtration [71].

In this method of filtration (Figure 2), two forces are involved: a shear force where the feed suspension flows parallel to the surface of the membrane; and a perpendicular force on the membrane surface generated by the trans-membrane pressure. Cross-flow mode is considered a mature mode of filtration, which is regularly used as a standard technique for liquid processing and concentration of product. There are numerous advantages of this mode including low energy consumption, an increase product yield, selective and consistent separation, low maintenance and no additives, flocculants, or chemicals required [69]. However, the perpendicular force is responsible for the formation of concentration polarization or gel layer, a build-up of retained material on the membrane surface [70].

## 5. Microfiltration

MF membrane process do account as a low-pressure membrane process. They are used for the retention of suspended material particles, closely resembling conventional coarse filtration. Membranes of a pore size range from 0.05–10 microns are used with typical operating pressures range from 0.5 bar to about 3 bar. This is ideal for separation of suspensions and emulsions. MF membranes moderately remove colloidal matter and suspended solids. Approximately 40% retention of organics could be achieved [72]. Promising results by using MF membranes have been also shown in defeating irregular and unpredictable increases of turbidity and NOM in complex systems such as karstic spring water [73].

### 5.1. Membranes for Microfiltration

In MF, when a symmetrical porous structure is involved, the complete membrane thickness may hinder transport. The thickness of MF membrane can extend from 10 microns to over than 150 microns. Nevertheless, majority of MF membranes are unevenly built up with a top-layer thickness in the order of 1 micron. Either organic materials (polymers) or inorganic materials (ceramics, metals, or glass) are used in the production of MF membranes. Several techniques are applied in the production of MF membranes including sintering, stretching, track-etching and phase inversion. The pore structure is usually symmetrical, with porosities as high as 80% [74].

### 5.2. Industrial Applications for Microfiltration

Microfiltration is a method of choice and is very well-established process for industrial applications where particles of a size greater than 0.1 microns have to be retained from a mixed solution. Examples (Table 2) include sterilization and clarification of numerous different genres of mixtures and solutions, in the food, feed and pharmaceutical industry which until recently were the main industrial applications [75]. MF is gaining great grounds in the water and wastewater industry, focusing in removing particles from water and wastewater, in sewage treatment but also in several other types of industries such as semiconductor fabrication that generate heavily polluted wastewater that needs extensive treatment due to toxic substances and metals [63]. 

Industrial applications of MF in the 20th century are often focused in the complete and safe removal of bacteria, parasites, and large particles to be achieved. MF however can only retain viruses to a significant degree under the appropriate conditions mentioned. The removal of parasites such as *Giardia* or *Cryptosporidium* has also been studied and it has been found the MF can be effective to an extent [76]. 

MF has been evaluated as an alternate treatment to the conventional treatment of groundwater (Florida, US) containing gaseous hydrogen sulfide (H_2_S) and compared with the conventional treatment basically utilized air stripping to remove H_2_S [77]. Using porous ceramic membranes to filter lake water has proven to be very useful for drinking water production. Suspended solids, microorganisms and algae were completely removed leading to a noticeable reduction in the chlorine demand which is necessary to render a hygienically safe transport and distribution network of water [78]. 

## 6. Membrane Manufacturing 

There is a wide range of available membrane materials that are employed in industrial process and more specifically in water and wastewater treatment. They vary more widely in chemical composition than in bulk morphology. The production of membranes (Table 3) can be by stretching, sub-atomic particle bombardment combined with etching and, in the case of ceramic membranes sintering [82]. Nowadays, most drinking water production membranes are made of polymeric material, due to the fact that they are significantly less expensive than membranes constructed of other materials. Membrane material properties greatly affect the design and operation of a filtration system, factoring aspects such as mechanical strengths or chemical reactivity. 

For example, membranes made of polymers that react with oxidants commonly used in drinking water treatment should not be used with chlorinated feed water, while a membrane with greater strength can obviously withstand greater TMP thus higher operational pressures can be applied. Likewise, a bi-directional strength membrane has the advantage of allowing cleaning operations to be performed from either the feed or the filtrate side.

Polymeric membranes have developed extensively giving rise to two types of membranes: isotropic and anisotropic. Nitrocellulose and cellulose acetate were first used in membrane manufacturing but were replaced relatively quickly by more sophisticated materials such as polyamide, polysulfone, polycarbonate and a number of advanced polymers [83]. 

On the other hand, membranes made of inorganic materials are generally having superior chemical and thermal stability. In the past, inorganic membranes, namely ceramics, were used for a single industrial application known as the enrichment of uranium hexafluoride (235U) by Knudsen flow through porous ceramic membranes [84]. This though has changed with inorganic membranes continuously gaining grounds with being able to be fabricated in various structures and pore sizes, especially as MF and UF membranes.

**Table 3:** Membrane manufacturing procedures and applications [85,86].

Membrane Materials	Manufacturing Procedure	Industrial Applications
Ceramic	Pressing, sintering of fine powders followed by sol-gel coating	MF, UF, aggressive (high concentration of acid and alkali chemicals for cleaning) and/or highly fouling media
Stretched polymers	Stretching of partially crystalline foil	MF, aggressive media, sterile filtration, medical technology
Track-etched polymers	Radiation followed by acid etching	MF, polycarbonate (PC) or polyethylene terephthalate (PET) materials. Analytical and medical chemistry, sterile filtration
Supported liquid	Formation of liquid film in inert polymer matrix	Gas separations, carrier-mediated transport
Integral asymmetric, microporous	Phase inversion	MF, UF, nanofiltration (NF), Gas transfer(GT)
Composite asymmetric, microporous	Application of thin film to integral asymmetric microporous membrane to produce TFC	NF, RO, pervaporation (PV)
Ion exchange	Functionalization of polymer material	Electrodialysis (ED)

### 6.1. Inorganic Membranes

In industrial applications, four kinds of inorganic materials have been used. These are ceramic membranes, glass membranes, metallic membranes (including carbon) and zeolitic a subcategory of ceramic membranes. Generally, metallic membranes are achieved by the sintering of metal powders (e.g., stainless steel, tungsten or molybdenum) and they have not gained much popularity, probably due to their high the cost a complex manufacturing. Glass membranes (silicon oxide or silica, SiO_2_) are primarily prepared by leaching on demised glasses and are rarely used to date [87]. 

Ceramic membranes have been widely applied in the industry, they are commercially available in numerous sizes and arrangements, although due to their high cost compared to their polymeric counterparts, their application has been limited until recently in the field of food, beverage and pharmaceutical industry [88]. 

However, their exceptional advantages, chemical and thermal stability as well as robust structural stability have attracted interest to their potential use in the treatment of waste streams [89]. Ceramic filters are fabricated using alumina, zirconia or zeolite, materials that withstand extreme pH, pressure conditions and high flux rates [90]. These characteristics facilitate effective cleaning with acidic or alkali solutions, indicating ceramic membranes as ideal candidates for processing complex effluent streams of sludge nature [80]. They have gained widespread popularity due to their specific properties as they can withstand high temperatures which are applied for instance in membrane reactors in which they contain the catalytically active sites and function as separation barrier as well [91]. Their uses regarding waste treatment have been expanded to the metal processing industry and surface engineering, in applications such as recycling and disposal of degreasing and rinsing baths; treatment of oil/water emulsions; recovery of heavy metals; cleaning of wastewater from grinding processes and treatment of wastewater from glass and glass-fiber production while regarding the environmental applications while environmental applications do include COD/BOD reduction; oil/water separation; recovery of pharmaceuticals and pesticides; retention of micro-organisms, heavy metals and radioactive substances; recycling of water from swimming pools and purification of the drain from sewage plants. MF is an economically viable alternative to traditional separation techniques such as centrifugation and rotary vacuum filtration.

Ceramic membrane configuration though still allows the deposition of particles in the inner side of the channels, forming a cake, which may hinder the permeate flux. 

MF on the mechanism of the sieve effect. Thus, micro-structure parameters (pore size, thickness, and porosity) of ceramic membranes affect permeate flux and retention. The resistance of ceramic membranes is measured via water permeability experiments [92,93]. 

### 6.2. Recent Developments of Membrane Materials

Since the main incentive in membrane formulation and fabrication research is fouling resistance, membranes possessing low affinity to pre-identified foulants in the feed suspension need to be developed. Natural foulants such as organic matter tends to be negatively charged; thus, membranes should possess a negative charge, in order to repel the foulant. The use of membranes for industrial process wastewater is limited by their resistance to extreme pH conditions and key organic solvents. Membranes for the development of biosensors [94] and molecularly imprinted polymeric membranes for separation of molecules are some of the most recent developments in membrane technology [95]. There is a wide range of chemical/physical mechanisms that can be used in membranes manufacturing, and that is one of the most attractive aspects of membrane processes. Therefore, successful applications will continue to be developed in the future. However, their industrial success will be governed by their advantages relative to other competing products and by their acceptance in the market.

## 7. Module Design and Configuration

Several factors are crucial to the overall process performance of a membrane such as its configuration, i.e., its geometry and the way it is mounted and oriented in relation to the flow of water. The configuration of optimum membrane is one that includes [96]:

- High membrane area to module bulk volume ratio
- High degree of turbulence for mass transfer promotion on the feed side
- Low energy expenditure per unit product water volume
- Low cost per unit membrane area
- A design that facilitates cleaning
- A design that also facilitates modularization

In general, the media of the membrane filtration is manufactured as hollow fibres or as flat sheet stock and then designed into one of many various types of membrane modules. Plenty of module designs are possible and all are based on two types of membrane configuration: flat or tubular. The modules of spiral-wound and plate-and-frame involves flat membranes while tubular, capillary and hollow-fiber modules are based on tubular membrane configurations [97]. Primarily, based on economic considerations, the module configuration, as well as the arrangement of the modules in a system is being chosen, bearing in mind many engineering parameters need to be employed to achieve this. Other features to consider include, separation type, density of the system, simplicity of operation, simplicity of cleaning, simplicity of maintenance, scale of the operation and the possibility of membrane replacement. 

### 7.1. Tubular Modules

**Figure 3:**
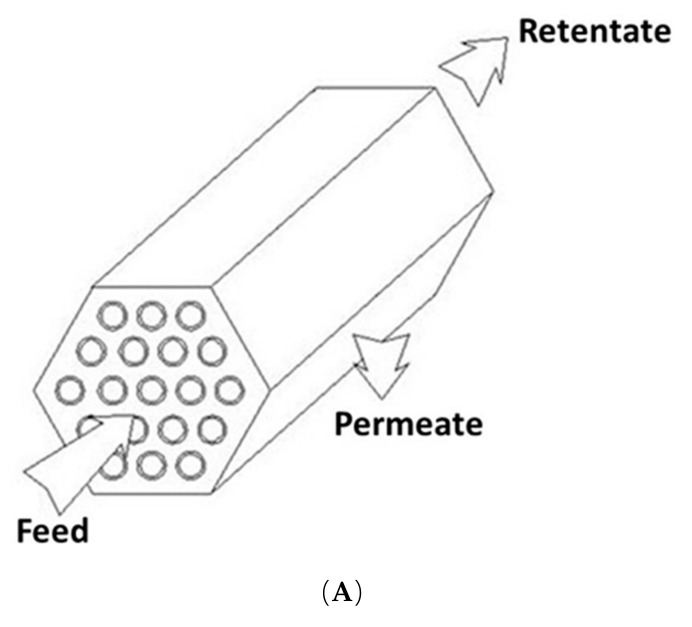

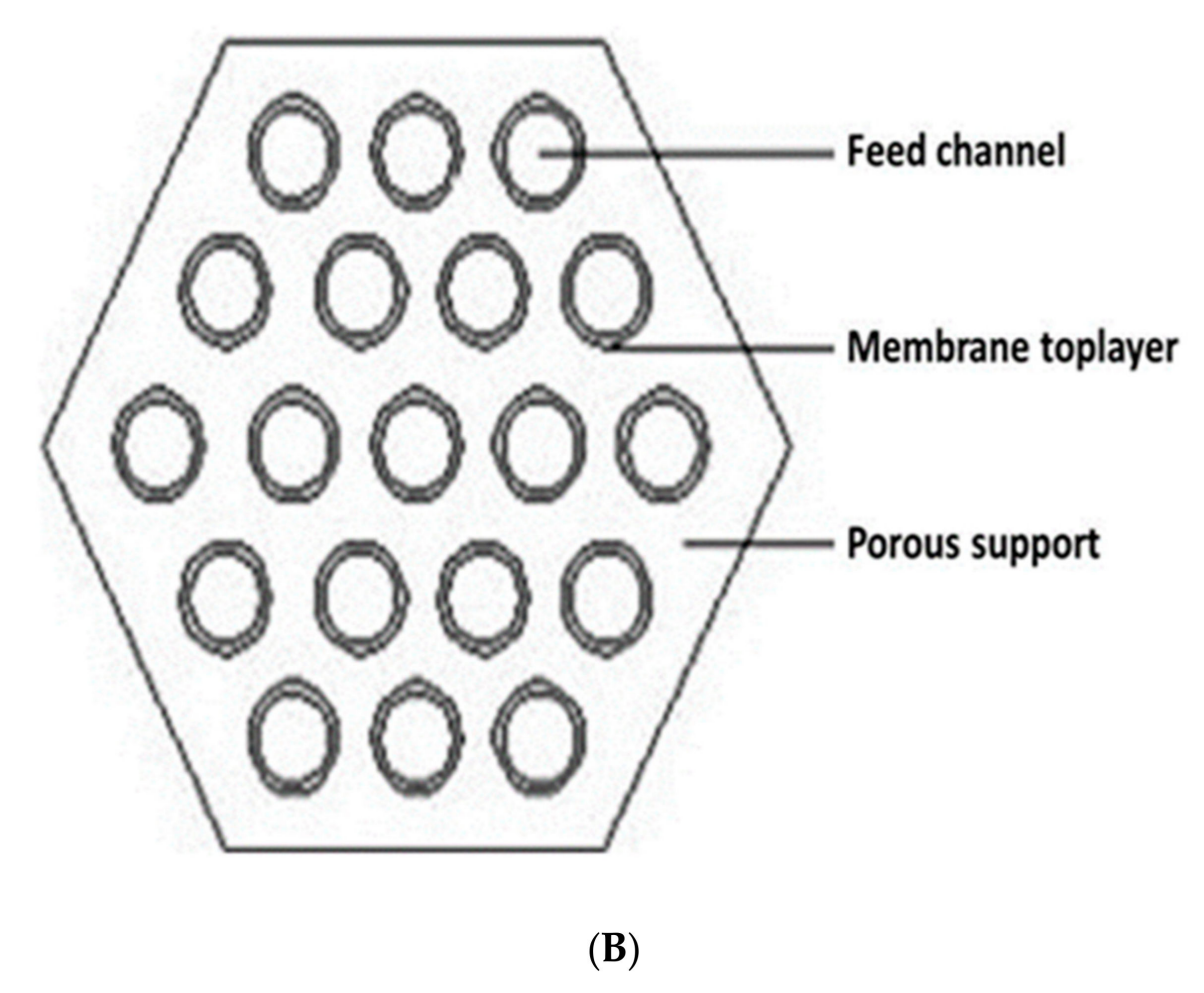
(**A**) Schematic drawing of a tubular module and (**B**) cross-section of monolithic ceramic module [99].

Tubular modules have the highest cost per unit area of all cylindrical membrane geometries; and they, also, provide a modest surface area to volume ratio (Figure 3). The feed solution feed through the center of the tubes while the permeate flows through the support layer into the module housing. 

However, the greatest turbulence promotion and the best access to the membrane surface could potentially be provided by tubular modules [98]. For high fouling matrices, both these features tend be preferred, therefore this configuration is popular. In general, the commercially available tubular modules are multi-channel, with mechanical support needed for polymeric tubes. Conversely, a lower packing density tends to occur in the ceramic tubular membranes due to their monolith construction. The ceramic tube wall is imposed for a lower limited thickness due to the fragility of the material, tending to increase the overall membrane resistance over a comparable polymeric membrane. Ceramics have also been considered as non-traditional material for tubular MF/UF membranes, even though there are currently not widely commercially promoted MF/UF systems for drinking water applications. The popular mechanical cleaning process for the large diameter fouled tube membranes utilizes foam balls, in which the foam balls process is used to wipe the inner surface of the membranes.

### 7.2. Hollow-Fiber Modules

**Figure 4:**
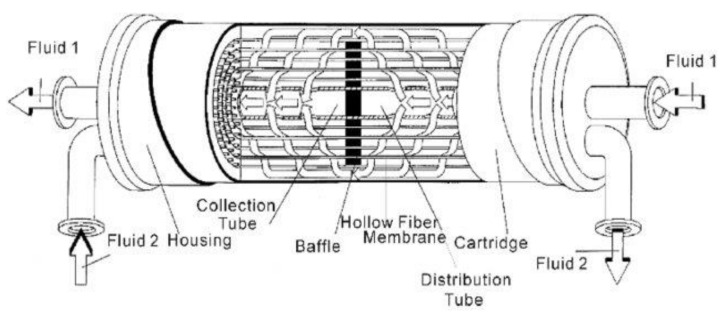
Demonstration of hollow-fiber membrane contactors for olefin/paraffin separation [102].

These modules possess the highest packing densities. These modules are simply constructed in principle. The bundle of fibers is bent in half, offering double the density and the ends plotted and exposed at one end of the tube into which they are inserted (Figure 4). The hollow-fiber modules are better used when the feed stream is relatively clean or free of very large particulates, as in gas separation, pervaporation, and seawater desalination. Consequently, there is a need for an effective pre-treatment prior to any process. The hollow-fiber modules are used in drinking water treatment applications and are mainly manufactured to accommodate porous MF or UF membranes and designed to filter particulate matter [100,101].

## 8. Current Developments in Module Configurations

Ceramic membranes have been at the epicenter of recent advances and research in membrane development and further growth in the area of water and wastewater treatment [103] with the scope being focused in results being geared towards creating a greater membrane area, without compromising mechanical strength. 

Commercial manufacturers of ceramic membranes and research engineers have focused for the most part on alternative materials and designs that are less complicated [104]. The challenge faced by suppliers is to cut the cost of ceramic membranes in order to make them further available and more economically competitive with the much less expensive polymeric membranes. 

In terms of method combinations, aeration combined with submerged membrane systems is yet another recent development. Submerged membrane systems, which were originally developed for membrane bioreactors (MBRs) in the late 1980s are large-area membrane filtration modules submerged in a tank where the permeate is removed at low TMP. They were considered as an alternative to conventional filtration systems. MBRs are an example of a hybrid process and in a submerged system turbulence is provided by coarse bubble aeration. It has also been presented by [105,106,107,108] that air-water two-phase flow can significantly improve membrane flux compared to single phase pumped liquid flow. Trials are still being conducted on static and moving turbulence promoters and turbulence promotion modifications to modules, including work that has been done over a ten-year period such as intermittent jets, where the feed is pumped coaxially through the membrane tube at fixed intervals through a nozzle [109]. It was noted up to 2.5-times increase in flux for bentonite suspensions. Pulsed flow has also been studied by several researchers [110,111,112,113]. In this case, pulses of flow are generated in the feed or permeate channel, creating changes in the velocity gradient. The use of inserts for turbulence promotion, especially in practical applications has gained more popularity [114].

## 9. Properties of Ceramic Membranes

Ceramic membranes possess various advantages; they are capable of separating mixtures physically, they are ecologically friendly due to their extended shelf life, and more favorable than other separation technologies, often no additives are required and there is no limitation to process temperature. Ceramic membranes can withstand high temperature filtration (up to 500 °C) and extreme pH conditions (1–14). They can be cleaned with aggressive chemicals, organic solvents, or hot water and steam. 

Ceramic membranes are chemically, mechanically, and thermally stable. They possess the ability of steam sterilization and backflushing; high abrasion resistance; high fluxes; high durability; bacteria resistance; possibility of regeneration; dry storage after cleaning.

Ceramic membranes have an asymmetric structure and consist of a coarse support, which is covered by several layers with decreasing pore size. Polymeric membranes are relatively unstable, but that problem has been greatly reduced or eliminated by using ceramic membranes [106]. The development of ceramic membranes has induced a moderate revival in the use of static turbulence promoters in cross-flow membrane filtration [115]. Running costs are limited by closed production cycles and continuous processes.

The high weight and considerable production costs of ceramic components are some of the disadvantages. However, costs are compensated for by a long service life. Polymeric membranes, on the other hand have limited stability (chemically, physically, and biologically), thus restricting the conditions of membrane processes applied.

## 10. Preparation of Ceramic Membranes

Ceramic membranes are a combination of a metal element (e.g., aluminum, titanium, silica or zirconium) with a non-metal in the form of an oxide, nitride or carbide. Ceramic membranes could be made from such materials, forming the major class of inorganic membranes, with aluminum oxide (α-Al_2_O_3_) or alumina and zirconium oxide or zirconia as the most important representatives. Commercial ceramic membranes are made by sintering or sol-gel processes them. The sol-gel is defined as the preparation of ceramic materials by preparation of a sol (solution), gelation of the sol, and removal of the solvent. The sol might be generated from organic or inorganic precursors, such as nitrates or alkoxides, and might consist of dense oxide particles or polymeric clusters [116]. The sol-gel process yields structures with pore sizes in the nanometer range. The pore structure can then be controlled by altering the manufacturing conditions involving both the staring materials and processing parameters on one hand, or by giving post modification treatments to pre-synthesized or commercialized membranes on the other hand [117]. To prepare ceramic membranes suitable for gas separation or reverse osmosis a further densification of the structure is required [118].

## 11. Ceramic Membrane Microfiltration

### 11.1. Gel Layer Formation and Control

Ceramic MF systems face numerous challenges with the main challenge faced is the possible formation of a gel layer on top of the membrane surface and caused by concentration polarization. Gel layer formation is considered as a reversible fouling mechanism [119]. This gel layer acts as a secondary membrane and could negatively affect the separation performance. It has been found that the gel layer resistance grows with higher solute concentration, possibly exceeding the resistance of the membrane and hence reducing product recovery. Furthermore, the overall yield could drop as a result of product loses in the gel layer. The concentration of the gel layer depends on the colloid size (or more commonly the colloid physico-chemical properties). Colloid size performs a role in the way the deposit is developed at the membrane surface [120]. A control of the gel layer builds up is needed to reduce the loss in flux permeability. 

The gel layer can be controlled and minimized if the ceramic membrane system is properly designed [121]. Effective designed systems would allow good control over convective forces such as tangential velocity and transmembrane pressure. Increasing the circulation flow would reduce the formation of the gel layer [122].

### 11.2. Technical Advances in Using Ceramic Membranes

The use of ceramic membranes offers several advantages which include long lifetime, easy cleaning, durability, operation in extreme circumstances, robustness. Over the last few years, extensive research has been carried out by institutions and membrane manufacturers to enhance the performance current ceramic membranes [123]. Improving the hydrodynamics of the system by promoting turbulence in the membrane boundary layer was of focus. Early trials include utilization of mechanical devices such as counter-rotating concentric cylinders or discs, introduction of gas bubbles, and use pulsed flow systems that reversed flow direction across the membrane.

## 12. Ceramic Membrane Fouling

**Figure 5:**
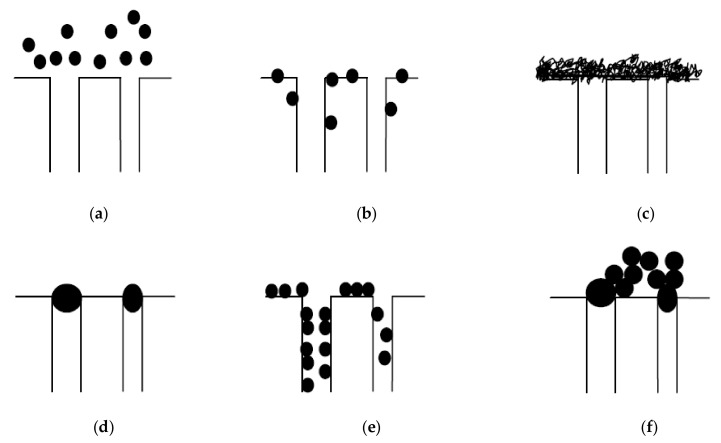
Mechanisms of membrane fouling [129,130]. (**a**) Concentration polarization; (**b**) Adsorption; (**c**) Gel layer formation; (**d**) Complete blocking; (**e**) Standard blocking; (**f**) Intermediate blocking.

The main hindrance to the widespread of pressure driven membranes is the occurrence of fouling phenomenon on the membrane surface. Membrane fouling can cause a decline in permeate flux and deteriorating of permeate quality [124]. It is generally caused by dissolved or suspended components in the feed. Such components include dissolved inorganic and organic components, bacteria, colloids, and suspended solids. These components can interact with either the membrane surface and/or the fouling layer. Membrane fouling can also be influenced by the hydrodynamics of the filtration process. Fouling is usually classified as reversible and irreversible [125]. Concentration polarization, gel layer formation and osmotic pressure are examples of reversible fouling [126]. These phenomena are easier to resolve than irreversible fouling. Examples of irreversible fouling include cake layer formation, adsorption, and pore blocking (Figure 5) [126]. There are three types of pore blocking, i.e., complete, standard, and intermediate [127,128].

The severity of fouling as well as the effectiveness of the cleaning method is revealed by the level of flux recovery. Concentration polarization and fouling are directly attributed to flux decline. Concentration polarization occurs when dissolved and/or colloidal materials concentrate on or very near the membrane surface while fouling is the gradual build-up of contaminants on the membrane surface [131]. Fouling of membrane is influenced by the filtration process hydrodynamics, the interactions between the membrane and the foulants in the feed stream, and between the fouling layer and the foulants [132,133]. Flux decline during MF can be very harmful on the economics of a given membrane operation and to tackle this problem, various measures are taken by organizations and companies [134,135].

### 12.1. Fouling Phenomena

**Figure 6:**
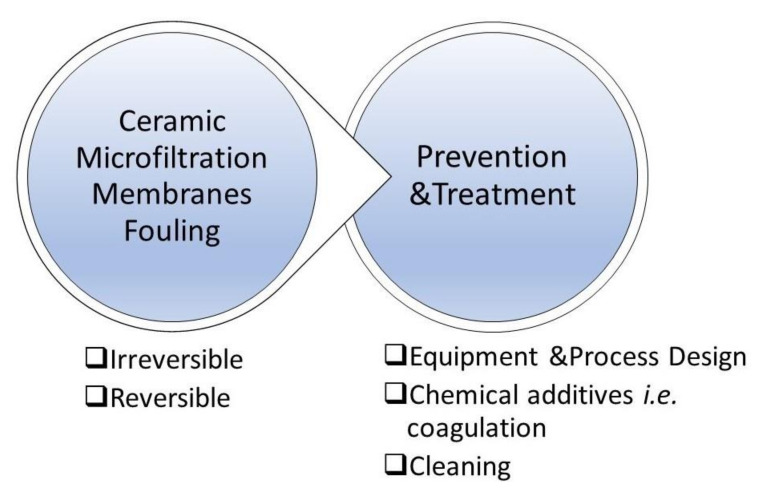
Membrane fouling prevention and treatment methods.

Understanding the fouling causing phenomena (Figure 5) and the mechanisms that cause them is pivotal to develop effective control methods and develop longstanding effective membrane operation processes (Figure 6). Particle adsorption and filtration-induced particle deposition are the two most important fouling phenomena in MF membranes, occurring through mechanisms such as concentration polarization and cake layer formation.

Particle adsorption on the membrane surface is usually irreversible and can occur even in the absence of filtration. In water treatment applications, the foulants are usually adhesive due to hydrophobic interactions, hydrogen bonding, van der Waals attractions, and extracellular macromolecular interactions amongst others [136]. Judicious choice of membrane material, size and properties can limit fouling caused by particle and molecule adsorption. For example, hydrophobic membranes usually have a stronger tendency to foul, particularly with proteins and yeast. These membranes do not facilitate water flow through their pores at average operating pressures (<1 bar). 

On the other hand, the particle deposition on the membrane surface is usually reversible non-adhesive fouling phenomenon [137]. Unlike irreversible fouling the membrane surface chemistry plays a weak role in the reversible fouling [138]. Accumulation of cell debris, organics, and other retained particles on top the membrane surface are examples of particle deposition reversible fouling.

### 12.2. Concentration Polarization

Concentration polarization is defined as the solute tendency to build up at the membrane-solution interface within a concentration boundary layer. The retained solutes can build up at the membrane surface and the concentration increases gradually. This concentration builds up will as a result produce a diffusive flow back to the feed bulk. 

### 12.3. Cake Layer

Cake formation is attributed to material accumulation on the surface of the membrane, which effectively leads to cake layer. The flow of permeate drives the particles to the membrane surface to form a cake layer on the membrane except if a very high shear rate is applied to prevent the cake layer formation. Long term fouling would be resulted by the accumulation of undetachable cake layer on the membrane surface [139,140,141]. In studies regarding river basin water clarifications [142,143], it has been found that the impact of cake formation on membrane fouling has more serious consequences than the adsorption of small substances with the membrane’s pores.

### 12.4. Fouling and Retention of Particles due to Natural Organic Matter (NOM)

Flux decline caused by natural organic matter (NOM) fouling is major problem in membrane filtration of brackish and surface water [144]. NOM interactions with membranes are main cause of NOM fouling [145,146]. The NOM mixture has a specific chemical nature. The charge, configuration, and chemical potential of NOM during filtration are affected by many operating parameters of the process such as pH, ionic strength, ion compositions, temperature, and pressure. 

pH plays an important role in the effectiveness of membrane processes to NOM removal. At pH values 6–8, NOM rejection is higher and NOM fouling is lower than pH higher than 8 [147,148]. This can be attributed to the increase in NOM molecular size and charge repulsion forces at a pH above 8. At higher pH, the water flux decreases which indicate that the charge of the membrane surface and pores plays a major role in the level of membrane flux and retention. The raise in pH causes the membrane surface and pores to become more negatively charged because of anion adsorption. This reduces the pores size and hence results in flux decrease and retention increase [149]. At low pH, NOM becomes very stable due to the fact that NOM molecules would contain approximately equal amount of carboxylate (COO-) and carboxyl (COOH-) groups which would lower the interactive forces between the fouling components and the membrane surface [150].

The adsorption of humic acids (HA) on the membrane surface affects the surface charge of the membrane and makes it more negatively charged. It is widely accepted that HA aggregates with larger molecular weight has higher adsorption potential [151]. This charge effect can be minimized by increasing the pH which causes electrostatic repulsion between HA and the membrane surface. Raising the pH can result in an increase in the hydrophilicity of HA.

The effect of the membrane pore size on the permeation of HA solutions has been previously evaluated [152]. It was found that concentration polarization is more prevalent in membranes with larger pore size. Additionally, the effect of pH is greater for the membranes with higher permeability, while in many cases the flux was non-linearly varied with varying pressures. 

The effect of NOM and humic substances on the deposition and retention of inorganic colloids by hydrophilic and hydrophobic membranes, have been previously investigated [153]. A number of process parameters have been tested including ionic strength, pH, calcium concentration, primary colloid size, and NOM concentration. The results showed that the particles with size close to the membrane pore size caused larger flux decline. In the presence of electrolyte solution and at pH values close to that of surface water, the membranes were able to fully reject the colloid aggregates and hence the flux decline was depending on the deposition on the membrane rather than the primary colloid size. This applies mainly to surface water with high turbidity by no organic content. The addition of organics into the electrolyte solution with aggregated colloids can cause the organics to adsorb on the aggregate surface and fouling increases compared to aggregates without organics. If the organics were first mixed with colloids and then mixed with electrolyte solution, charge stabilization of the colloids can occur due to adsorption of the organics on the colloid surface. As a result, rejection falls to almost zero and fouling becomes fully dependent on primary colloid size. Rejection can be increased by destabilization of the colloids using calcium. 

In membrane processes, increasing feed flowrate increases both recovery and permeate flowrate up until an optimal feed flow is attained, and then recovery starts to decrease [154]. According to some researchers a low flux–high recovery process is more appropriate than high flux–low recovery approach for direct application on seawater [155] as well as highly fouling surface water due to possible severe membrane fouling and plugging of fibers [156]. The High flux–high recovery system can cause a raise in TMP. The advantages of operating with higher flux have to be balanced against associated disadvantages such as increase in chemical cost and backwashing. 

Major fouling challenge comes from NOM fraction consisting small, neutral, and hydrophilic compounds [157]. It has been observed [158,159] that permeate flux decline comes mostly from hydrophobic fraction of NOM whereas the hydrophilic fraction caused much less fouling. NOM with larger molecular weight fraction contributed to the formation of the fouling layer since the size of these NOM is usually larger than the membrane pore size and hence cause surface fouling.

A number of investigations found that in MF, cake formation and pore plugging were responsible for membrane fouling as they reduce pore size and increase retention. Internal pore adsorption of calcium-organic flocs reduces the internal pore diameter and consequently increases rejection. The characteristics of membranes rejection function do not depend on initial membrane characteristics as much as the fouling state of the membranes and the nature of the foulants [160]. 

The size and shape of macromolecular solutes and operating pressure play a significant role in membranes fouling [161]. Colloid stability greatly affects fouling when lowering colloid stability worsens its degree and makes thicker deposits on the membrane surface. It was also found that increasing the shear rate helps to reduce concentration polarization of HA in MF membranes. The molecular diameter was found to be more useful for describing the membrane sieving mechanism.

Many studies found that humic substances cause irreversible fouling in MF membranes. For example, aggregate HA was responsible for the initial stage of fouling in a hydrophilic MF membrane. The effect of HA and fulvic acid (FA) was studied [161] on membrane performance and found that HA was responsible for a 78% decline in flux compared to only a 15% decline with FA, and it has been hypothesized that this could be due to the HA’s aromatic and hydrophobic properties, adsorptive behavior and greater MW that led to tendency to foul. 

## 13. Methods Employed to Increase Retention and Reduce Fouling

### 13.1. Integration of Coagulation with Membrane Filtration

Coagulation, in water and wastewater treatment, is the destabilization of NOM solutions using coagulants. Coagulants can be classified into two main categories, i.e., metal coagulants, such as aluminum sulfate, Al_2_(SO_4_)_3_·16H_2_O (Alum), and polymers such as poly-diallydimethylammonium chloride (PDADMAC). Coagulation is managed to overcome the factors that promote NOM solution stability and form agglomerates or flocs. Flocculation in other words is the process of whereby destabilized particles, or particles formed because of destabilization, are induced to come together, make contact, and hereby form larger agglomerates [162]. 

### 13.2. Coagulation

Coagulation/flocculation processes are principally used for the removal of colloidal material, which change color and cause turbidity, as it cannot be removed easily from water by means of the usual conventional separation methods including sedimentation and filtration. Coagulation has been as a pre-treatment used to remove small particles from aqueous suspensions prior to membrane filtration or conventional sedimentation of surface water. 

Particulates of organic nature strongly interact with cationic additives, particularly metal coagulants hydrolysers and cationic polyelectrolytes. Studies [163] attached much importance to the initial water quality as well as treatment conditions (initial turbidity, initial pH, coagulant dose, flocculation time and pre-ozonation dose) that affect DOM removal during alum coagulation. The study provided an elaborate analysis about the effects of the characteristics of dissolved organic matter (DOM) on its removal, namely: HA content, molecular weight distribution and the HA fraction carboxylic acidity. The methodology of coagulant addition depends on the rate of the reaction between the coagulant and the soluble part. Coagulant reactions are very fast with some completing within a few seconds following coagulant addition. There are various types of coagulants, organic and inorganic such as aluminum sulfate, ferric chloride, cationic polymers (PDADMAC) [164].

Various optimal doses were determined for several water and NOM qualities resulting from the coagulation processes sensitivity, for example, the optimal dose is 70 mg/L regarding surface water treatment with aluminum sulfate. The optimal coagulant dose is determined by a seasonal and day to day variations in the raw water chemical and physical conditions including pH, NOM, and temperature. The rationale behind the coagulation optimal dose is that higher doses may lead to an increase in the settled water residual turbidity, while low doses may lead to a substantial reduction of water residual turbidity. For coagulant concentration, there is a threshold under which plant operation may be compromised. Hence floc growth must proceed up to some critical floc size before challenging membrane filtration, otherwise the membrane will be partially irreversibly clogged due to the flocculant’s solids. The most important parameters for optimal treatment were the chemical type of coagulants and their dosage followed by the water pH and last the test solution [165]. Optimal pH values vary among different coagulants. Thus, a pH of 5.5 is regarded as optimal to coagulate HA found in river water while the optimal pH for an alum system appears to be in the range of 7.5 to 8 based on cake formation. 

Due to their lower apparent carboxylic acidities and higher molecular weights, humic acids are preferentially removed by means of alum coagulation. Coagulation is obviously enhanced when the organic content is relatively high in water [166]. Moreover, the coagulant multi-valency i.e., Al^3+^ and Ca^2+^ ions could lead, as expected, to larger molecular particles [167]. Experiments indicated that removal of organic matter by coagulation is directly proportional to the constituent’s molecular weight. However, the interaction between for example alum and HA involves other factors including formation of complexes, precipitation, charge neutralization and adsorption. Increasing the dosage, the major mechanisms of HA removal will be expected to shift from formation of complexes, charge neutralization and precipitation to adsorption [168].

Polymer coagulants have removed the hydrophobic NOM fractions effectively while highly charged polyelectrolyte led to better removal of humic acid (around 90%) [169]. The cationic polymers performance was significantly enhanced with increasing molecular weight and charge density indicating synergistic effects. In cases where the polymer added is capable of bonding to the colloidal particles surface, it can then behave like a flocculent, thus making these materials often quite target specific [170]. The retention produced by polymers is lower than the retention produced by alum (96% vs. 99%), but these polymers have the advantage of producing less compacted sludge [171]. Flocculation improves when the polymer molecular weight is increased, which is probably due to better bridge formation. If the polymer concentrations are low, the number of polymer molecules adsorbed per particle would be small. As the polymer and the supernatant possess opposite charges, the polymer adsorbs with a flat configuration, thus making the possibility of bridging limited. Hence, to make the number of adsorbed polymer molecules per single particle higher, the polymer concentration is needed to be increased [172].

### 13.3. Integration of Coagulation with Membrane Filtration

Membrane separation has the advantage, over conventional clarification techniques, of reducing the flocculation time to a great extent, thus allowing the construction of a compact space wise plant [173]. Water with high organic matter load, can be better treated by coagulation combined with microfiltration filtration or microfiltration alone. In an integrated or hybrid process, the organic matter content in water as well as its turbidity can be considerably decreased down to the level suitable for drinking [174].

**Figure 7:**
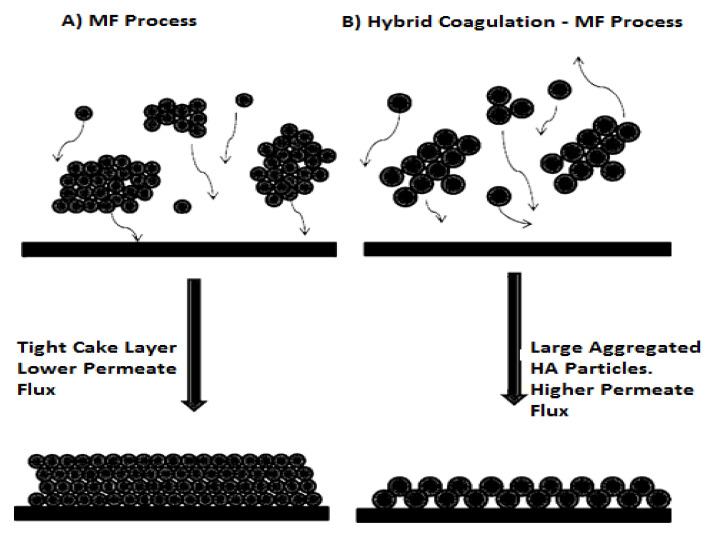
Schematic of cake layer formation for MF (**A**) and hybrid coagulation–MF processes (**B**) [177].

When MF is used for humic acids containing water treatment, a tight cake layer might be formed on the membrane surface [175]. This tight cake layer reduces the permeate flux. Therefore, for water treatment, it is more desirable to combine the use of coagulation and membranes filtration processes because the coagulation give HA the opportunity to join with other particles present in water before HA reaches the membrane surface [176]. Coagulation aggregates the HA particles and produces larger particle sizes that cannot block membrane pores, thus, giving the water a chance to pass through the MF membrane (Figure 7).

Before the treatment of NOM in wastewater, pre-treatment agents including polymers and metal coagulants must be carefully selected because coagulants can damage the membrane or enhance fouling. Thus, these coagulants (polymers and metal coagulants) can then become a source of fouling [178].

Hollow-fiber cross flow coagulation and MF were used to treat secondary oxidation in a pond effluent. Using an optimal dose of Moringa oleifera (a natural coagulant of plant origin), the performance of MF and coagulation were investigated. MF combined with coagulation gave better flux performance as well as lower rates of fouling. However, no significant influence was observed on the values of biochemical oxygen demand (BOD_5_), chemical oxygen demand (COD), alkalinity, total solid (TS), volatile suspended solid (VSS), turbidity and pH values in the filtrate when microfiltration was combined with coagulation using Moringa oleifera [179]. Various chemicals were studied in terms of their effectiveness on fouling reduction and filterability in membrane bioreactor mixed liquors. The results indicated that all the tested cationic polymers, chitosan and starch reduced fouling rates to a great extent and improved permeability values. Based on the lab-scale tests that were conducted, cationic polymers performed better relative to the other additives as they showed a steady fouling control, and performance persisted against minor dosing variations. However, in the case of other additives, filterability was considerably affected by higher and lower dosing. Generally, cationic polymers provided reductions in fouling rates in the range of 74–96% [180]. 

Studies indicated considerable increases in steady state flux when coagulation was combined with MF using cationic polyelectrolytes. Considerable improvement also occurred in effluent quality, in terms of COD removals and turbidity, when MF was integrated with coagulation. COD removal was improved up to 60% and turbidity removal by 75% when MF was coupled with coagulation, which showed much better performance than when MF was used alone [181]. The fouling rates were relatively low for both the weak and strong hydrophobic fractions as well as in the case of the charged hydrophilic fraction. Partial NOM removal was reported when either an adsorbent or a coagulant was used, but the use of a coagulant is favoured over the use of an adsorbent as it has the advantage of reducing the rate of fouling [182]. The use of FeCl_3_ at a pH of 5.5 as a coagulant did not increase NOM retention. When humic water was filtered in an ion exchange process following the addition of ferric iron, flux reduction significantly increased. Although the ferric iron was thought to preclude the transmission of NOM by means of complex formation, it did not live up to this expectation because of the relatively large pore size of the membrane [183]. 

Other studies [183] indicated that NOM concentration can be reduced by means of coagulation pre-treatment using metal ions, but such pre-treatment did not prevent or reduce membrane fouling. 

Flocs, another important factor which is resistant to shear stress during inline coagulation, was investigated [184]. Flocs resulting from the metal coagulant proved to be delicate and easily broken, because of shear stress [185]. The flocculation mechanism is affected by the polymer molecular mass which is also important for the processes of hybrid coagulation-membranes. Flocs resulting from the bridging mechanism are markedly stronger than the ones that result from charge neutralization. On the other hand, the cake that forms on the membrane top surface after coagulation is characterized by less hydraulic resistance [186] and hence can be backwashed easily. This possibly results from the less sticky cake layers produced by metal coagulants like alum. Organic compounds interact with the hydrated aluminum attached on membranes which is known as residual alum, and they can be cleaned easily [187].

### 13.4. Turbulence Promoters

Cross-flow membrane filtration uses many hydrodynamic methods to improve mass transfer, and the simplest way to reduce membrane fouling by creating turbulence, is to increase cross-flow velocity. In this simple method, the turbulent shear stress mixes the fluid with the bulk flow in the boundary layer. However, its use is limited by the large pressure variations along the membrane length and the high processing costs. Another simple method to speed shear stress, is the use of static turbulence promoters near the membrane surface [188]. 

This requires attaching the static promoters to the design and inserting non-static promoters to the feed stream. The membrane surface may suffer some damage due to these particles which may bring a negative effect to the concentrate treatment. The results of these studies along with the development of ceramic membranes, prompted the study of static turbulence promoters use in cross-low membrane processes, especially helical-shaped membranes [189]. Turbulence methods are among many methods used to reduce or prevent fouling on membrane surfaces. Turbulence promoters are constructed in many sizes and shapes including metal grills, static rods, cone shape inserts, spiral wires, and doughnut and disc-shaped inserts. Most promoters are of helical design, in order to create flow regimes, force the generation of dean vortices, demonstrate good mixing and reduce localized concentrations and polarization [190]. The inserts also create vortices, which in turn improve fluid mixing and reduce concentration and polarization effects [191]. Some studies reported filtration flux increases of up to 6–10-times, which were directly linked to increases in wall shear stresses on the membrane surface [192].

Turbulence promoters’ function by creating fluid instabilities, which induce turbulence through the static mixers and feed spacers. These fluid instabilities are used to disturb foulants, while channels with irregularities are used to induce mixing at the membrane and solution interface [193]. Another method used to improve the filtration process is to promote turbulence using stamped membranes and baffles (helical shape) [194], inside tubular ceramic MF membranes, and obtaining increase in the permeate flux 1–4 times over the smooth-surface membrane. Collapsible tubes pulsation generators have also been used, interrupting periodically the cross flow across the system, that improved flux significantly [195].

Other techniques have been used to disturb the boundary layer and improve cross-stream mixing including inserts in flow channels and variation in the filtering surface geometry . Intermittent jets as well as pulsatile flows have also found to be effective. A rotating blade has also been used in a flat plate module to increase shear stress rates [196]. Dean vortices, Taylor vortices and pulsatile flows in passages, all of which are designed to generate vortices, have all been found to reduce concentration polarization and increase filtration fluxes [197].

### 13.5. Electrical Field

Theoretically, several alternative forces can be used to improve the performance of MF and reduce fouling resulting from charge interactions between the membrane and charged solutes. Among these alternatives, the use of electric field has been further investigated and has been studied to reduce fouling emanating from charge interactions between the membrane and charged solutes [198]. Electro-filtration is among the techniques developed to counter residual cake formation on the membrane surface using an applied electric field. In this process, the applied electrophoretic force restricted the build-up of solutes on the surface of the membrane. The permeation rate is also improved via the filter cake because of electro-osmosis which is a secondary electro kinetic phenomenon [199]. The advantage of this method is that low cross-flow velocities can be used in the industrial context by applying this method [200]. Cross-flow velocity could be lowered to 0.1 m/s, which directly reduces the heat inputs, pumping costs and improves shear streams. However, power consumption and the corrosion of electrodes have limited the commercial success of this process. Problems associated with this process like the above, can be reduced by pulsing the electric field, which can be done by applying a potential at intervals. The degree of flux improvement primarily depends on the solute’s particle or molecule size, surface charge, and the imposed field gradient magnitude. As the process uses a DC electric field, it also has the disadvantage of gas evolution by electrolysis [201]. 

Power consumption and other problems resulting from the continuous application of electric fields, can be solved by pulsing the electric field, which may be done by on and off switching of the applied potential at regular or irregular intervals as may be appropriate. Electric fields use has also been found to be efficient in preventing fouling and increasing the permeate flux in the synthesis of dyes. Researchers utilized stainless steel microfilters, as filters and cathodes, and applied a continuous potential. In the wake of this development [202] found that it is possible to conduct electrolysis on the feed liquid as suitable mechanism to reduce fouling. In this process hydrogen gas evolves at the cathode and continually scatters the material that deposits. These authors introduced electric field short pulses in order to disperse the deposits that build up on membranes as they are electrically conductive. Over the years, other methods that employ electrically enhanced filtration have appeared in research papers [203].

### 13.6. Ultrasonic Field

Researchers have also studied the use of ultrasonic fields as a means to reduce membrane fouling. Ultrasound waves can be passed through a suspension in order to disperse its particles, reduce its viscosity, change its particle surface properties, and cause cavitation. Although dispersion can potentially increase fouling by means of the formation of highly resistant membrane deposits, it enhances permeation by combining cavitation with relative movement between solid and liquid phases. It is also possible to increase the permeate flux by the simultaneous application of electric and ultrasound fields. Both fields have been shown to limit fouling when applied individually, but improvement due to the ultrasonic field is expected to be minimal [204].

### 13.7. Backwashing (Backflushing) 

Backwashing is an often-compulsory step for sustainable filtration, with continuous research being done to improve the efficiency and performance of backwashing. It is a timed procedure, commonly involving the purging of the membrane pores in time intervals with either air and water or cleaning reagents. Usually it is applied when the permeability rate of the membrane starts to drop due to the formation of a cake layer accumulated on the membrane surface [61]. Pores plugged or fouling, are considered as a significant drawback in the MF membrane process. All types of reversible foulants including organic chemical agents such as NOM, particles fouling, and biofouling can decrease the rate of permeability of the membrane [205]. During the backwash process, some of the weakly bonded foulants are flushed out and the clusters get fragmented, however it has been found that microscopic impurities can still be found on membrane pores past backwashing [205]. There are two main backwashing methods, physical and chemical backwashing. In physical backwashing, only water and air are used during the flushing process, whereas in chemical backwashing, a chemical of choice is used while the backwashing process is taking a place. In spite of the importance of chemical cleaning, removing fouling with cleaning is usually costly as it requires the complete shutdown of the process the membrane is involved, and affects to an extent the lifespan of the membrane itself, as it often involves strong, highly concentrated chemicals that can cause damage in the structural integrity of the membrane itself if not used judiciously. Introducing steam instead of increasing the chemical concentration during the membrane cleaning process can be in certain cases more beneficial [206].

## 14. Membrane Cleaning

The product flow, during long membrane performance, constantly decreases due to progressive adherence of different foulants to the membrane’s surface, the matter which raises hydraulic resistance in the membrane module and diminishes its active surface. To restore the initial flux levels, chemical regeneration procedures may be performed to remove the build-up of foulants, in the cleaning process. Cleaning may be defined as a process where material is relieved of a substance that is not an integral part of the material [207].

The goal of cleaning is to acquire a physically clean structure. A considerable body of research proposed cleaning procedures for fouled membranes, as well as mild cleaning regimes and environmentally friendlier cleaning procedures, such as regimes in which purified enzymes and detergents are used in order to remove biologically derived foulants that foul polymer membranes. Attractive alternatives have been proposed to replace classical cleaning regimes such as the use of an enzymes as standalone processes or combined with biodegradable detergents [208]. Enzymes are regarded as ideal cleaning agents due to their highly specificity for the reactions which they catalyze as well as the substrates they interact with. Generally, cleaning is carried out in different physical, chemical, and biological forms.

In the case of chemical cleaning, the first step is to find suitable materials to be used as cleaning agents [209]. The choice of the suitable materials rests on the feed composition and the layers precipitated on the membrane surface which is performed in most cases by trial and error. The choice of the cleaning solution does not depend on the foulant type only, but also on the membrane compatibility with the cleaning solution at the cleaning temperature [210]. The wrong choice of a cleaning agent can adversely compromise the performance of the membrane. The selected cleaning agent must be safe, chemically stable, cheap, and easily washable with water, as well as capable of dissolving most of the precipitated fouling materials on the membrane surface without damaging it [211]. Poor permeate flux due to irreversible fouling can only be recovered by chemical cleaning or by mechanical backwashing or both. Cleaning agents (Table 3) usually belong to the categories of bases, acids, enzymes, surfactants, and disinfectants and combinations of these categories [212]. 

Appropriate selection of cleaning chemicals entails a thorough understanding of the foulant’s chemical properties. Chemical effects are now largely understood. The higher the foulant molecular weight and charge ratio, the greater is the fouling rate by potable water. Fouling is also increased by the presence of divalent cations [213]. An increase in the electrostatic potential of the cleaning medium due to higher charge density, polarity, or pH, restrains the attraction forces and thereby increases cleaning efficacy [213].

### 14.1. Cleaning Reagent Performance

Mass transfer, is the second defining cleaning mechanism, is believed to be the main barrier to effective chemical cleaning. The chemical agent is prevented from reaching the foulant unless sufficiently high concentrations are used to overcome the attraction forces [214].

Several researchers [215] have proposed six steps to summarize membrane chemical cleaning process, as follows:

- Bulk reaction (hydrolysis and other) of cleaning reagent as the cleaning in place (CIP) is introduced.
- Cleaning agent is transported to membrane surface.
- Cleaning agent transits through foulant layers to membrane surface.
- Cleaning reactions solubilise and detach foulants.
- Waste cleaning agent with suspended foulants transported to interface.
- Finally, transport of waste matter to the bulk solution from retentive side of membrane.

**Figure 8:**
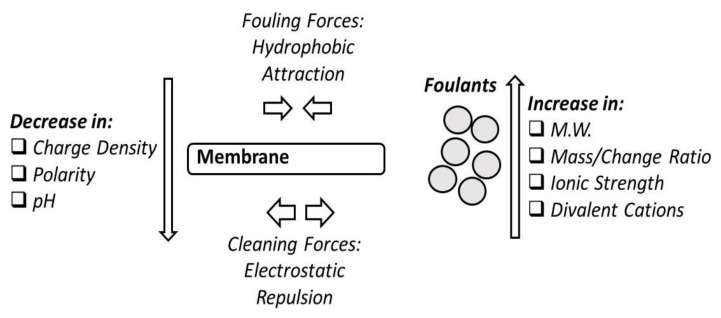
Schematic stage model for solution transport [216].

Based on the electrostatic equilibrium model (Figure 8), forces that retain the foulant at the membrane surface are minimized during cleaning as a step towards its physical removal. Hence the selection of the cleaning agent should depend on the nature of the foulant, whether it is organic or inorganic, or acidic or basic, as well as the charge state. Table 4 shows the physic-chemical mechanisms that describe the functioning of the most commonly available membrane cleaning agents that are used to clean potable water.

### 14.2. Caustic Soda

Sodium hydroxide (NaOH) solutions are used when membrane chemical resistance is a problem. Usually, 1% NaOH concentration is used at the pH levels of about 11–12, or less. It interacts with the weakly acidic organic matter, usually with the carboxylic and phenolic functional groups. It also aids breaking of polysaccharides and proteins into smaller molecules of sugars and amides [217]. NaOH also saponifies fats and solubilizes proteins. A large body of research demonstrates the efficiency of NaOH in washing away whey protein deposits from MF membranes [218]. It can also expand NOM molecules, thus allowing higher mass transfer and movement of the cleaning agent to the membrane surface. The hydroxide is also thought to remove inorganic colloids and silicates by increasing solubility and electrostatic repulsion. More permeate could be recovered when NaOH is used at the threshold value concentration which varies for different foulants and membrane materials and degree of fouling [219].

### 14.3. Oxidants

Sodium hypochlorite (NaOCl) and hydrogen peroxide (H₂O₂) are among the oxidants that are used in membrane cleaning procedures. Sodium hypochlorite is very common, yet there is no general agreement on its preferable use. Oxidants degrade NOM functional groups to ketonic, carboxyl and aldehyde groups which makes them readily hydrolysable at high pH levels. This could explain why when alkaline cleansing agents combined with oxidants, the oxidants become more effective, especially where organic foulants dominate [220].

### 14.4. Acids

Acids are used to remove multivalent cationic particles found in hard water such as salts and metal hydroxides. Nitric acid (HNO₃) has been shown to solubilize inorganic materials that contain bases such as calcium phosphate (Ca_3_(PO_4_)_2_). Rinsing inorganic membranes such as zirconia with HNO_3_ gives higher water fluxes [221]. Mineral acids, especially hydrochloric acid (HCl) and sulfuric acid (H_2_SO_4_), are in common use because of their low costs. They are effective for both cleaning in place (CIP) and chemical enhanced back flush at pH of down to 1.0, acids are used more commonly for the removal of mineral scaling.

## 15. Conclusions

Ceramic MF membranes are continuously gaining grounds as parts of the daily systematic water and wastewater treatment. Membranes should be used judiciously can offer high productivity of pollutants retention and low operational cost compared to other competing technologies, since there is no phase change of water and minimal or no use of chemical additives. Ceramic membranes have high resistance to extreme operating conditions and cleaning protocols. This allows longer service lifetime and highly efficient filtration performance. The following though can be summarised:

- Fouling remains the toughest hurdle regarding to the even greater use and implementation of the membranes in the industry.
- Fouling is a complex multifactorial phenomenon which although there is a much higher level of understanding today comparing to the past, further research is needed for its further alleviation.
- Numerous ways of addressing fouling are been investigated and implemented in the industry including hydrodynamics, testing different materials of fabrication, testing different pre-treatments, i.e., coagulation, hydrodynamics and cleaning with different agents and techniques.
- Although several other highly sophisticated methods for prevention of fouling such as ozonation have been tested, coagulation remains the widely applied option.
- As fouling prevention mechanisms such as intermittent cleaning with agents and other relevant cleaning strategies do remain the main method to address the occurring problem in industrial scale.

## Figures and Tables

**Table 2:** MF industrial wastewater treatment.

Membrane Type	Industrial Wastewater Application	Reference
**Ceramic Microfiltration**	Marcellus shale flowback water	[69]
	Dairy wastewater	[70]
	Aquatic humic substances and algal organic matter	[71]
	Aqueous bacterial cell debris	[72]
	High-turbidity water (overflow)	[73]
	Activated sludge	[74]
	Carbonated and filtered remelt syrup	[75]
	Oily wastewater treatment	[76]
	Municipal wastewater	[77]
	Oil-water emulsions	[78,79,80,81]

**Table 4:** Membrane cleaning agents and their acceptable physical-chemical mechanisms.

Cleaning Agents	Chemical	Reactions
**Base**	Caustic Soda (NaOH)	Hydrolysis and solubilisation, saponification
**Oxidants**	Hypochlorite (HOCl), Hydrogen Peroxide (H_2_O_2_)	Oxidation and disinfection
**Acids**	Hydrochloric Acid (HCl), Sulfuric Acid (H_2_SO_4_), Nitric Acid (HNO_3_)	Solubilisation
**Acid chelate**	Citric acid	Chelation
**Alkaline chelate**	EDTA	Chelation
**Surfactants**	Proprietary	Emulsifying, dispersion and surface conditioning

## References

[1] Www.Prb.Org. https://www.prb.org/Populationtrendsandchallengesinthemiddleeastandnorthafrica/.

[2] Agriculture & Water. https://www.saudiembassy.net/agriculture-water.

[3] Water Resources. https://www.saudiembassy.net/water-resources.

[4] Alkhudhiri A., Darwish N.B., Hilal N. (2019). Analytical and Forecasting Study for Wastewater Treatment and Water Resources in Saudi Arabia. J. Water Process Eng..

[5] El-Ghonemy A.M.K. (2012). A small-scale brackish water reverse-osmosis desalination system used in northern Saudi Arabia: A case study. Renew. Sustain. Energy Rev..

[6] Corominas L., Flores-Alsina X., Snip L., Vanrolleghem P.A. (2012). Comparison of different modeling approaches to better evaluate greenhouse gas emissions from whole wastewater treatment plants. Biotechnol. Bioeng..

[7] Bis M., Montusiewicz A., Piotrowicz A., Łagód G. (2019). Modeling of Wastewater Treatment Processes in Membrane Bioreactors Compared to Conventional Activated Sludge Systems. Processes.

[8] Zularisam A.W., Ismail A.F., Salim R. (2006). Behaviors of natural organic matter in membrane filtration for surface water treatment—A Review. Desalination.

[9] Barakat M.A. (2011). New trends in removing heavy metals from industrial wastewater. Arab. J. Chem..

[10] Van Der Bruggen B., Vandecasteele C., Van Gestel T., Doyen W., Leysen R. (2003). A review of pressure-driven membrane processes in wastewater treatment and drinking water production. Environ. Prog..

[11] Edzwald J.K. (2000). American Water Works Association (AWWA) Water Quality & Treatment: A Handbook on Drinking Water.

[12] Zacharof M.P., Lovitt R.W. (2014). The filtration characteristics of anaerobic digester effluents employing cross flow ceramic membrane microfiltration for nutrient recovery. Desalination.

[13] Zacharof M.P., Mandale S.J., Oatley-Radcliffe D., Lovitt R.W. (2019). Nutrient recovery and fractionation of anaerobic digester effluents employing pilot scale membrane technology. J. Water Process Eng..

[14] Department of Energy & Climate Change (2011). The Carbon Plan: Delivering our Low Carbon Future Presented to Parliament Pursuant to Sections 12 and 14 of the Climate Change Act 2008 Amended 2nd December 2011 from the Version Laid Before Parliament on 1st December 2011.

[15] DECC (Department of Energy and Climate Change Website) (2014). Increasing the Use of Low Carbon Technologies. https://www.gov.uk/government/policies/increasing-the-use-of-low-carbontechnologies.

[16] (2013). Waste Management Plan for England.

[17] Department for Environment, FARA (2012). Wastewater Treatment in the United Kingdom—2012 Implementation of the European Union Urban Waste Water Treatment Directive—91/271/EEC.

[18] (2014). Review of Waste Policy and Legislation EU Waste Framework Directive 2008/98/EC, the Landfill Directive 1999//31/EC and the Packaging and Packaging Waste Directive 94/62/EC.

[19] Rice J., Wutich A., Westerhoff P. (2013). Assessment of De Facto Wastewater Reuse across the US: Trends between 1980 and 2008. Environ. Sci. Technol..

[20] Richardson B. (2012). From a fossil-fuel to a biobased economy: The politics of industrial biotechnology. Environ. Plan. C Gov. Policy.

[21] Abadi S.R.H., Sebzari M.R., Hemati M., Rekabdar F., Mohammadi T. (2011). Ceramic membrane perfor-mance in microfiltration of oily wastewater. Desalination.

[22] D’souza N.M., Mawson A.J. (2005). Membrane Cleaning in the Diary Industry: A Review. Crit. Rev. Food Sci. Nutr..

[23] Xu W., Chellam S., Clifford D.A. (2004). Indirect evidence for deposit rearrangement during dead-end microfiltration of iron coagulated suspensions. J. Mem. Sci..

[24] Bouwer H. (2002). Artificial recharge of groundwater: Hydrogeology and engineering. Hydrogeol. J..

[25] Qadir Q., Bahri A., Toshio Sato T., Al-Karadsheh E. (2010). Wastewater production, treatment, and irrigation in Middle East and North Africa. Irrig. Drain. Syst..

[26] Qadir M., Wichelns D., Raschid-Sally L., McCornick P.G., Drechsel P., Bahri A., Minhas P.S. (2010). The Challenges of Wastewater Irrigation in Developing Countries. Water for Food Faculty Publications. Agric. Water Manag..

[27] Rice J., Wutich A., Westerhoff P. (2015). Spatial and temporal variation in de facto wastewater reuse in drinking water systems across the U.S.A. Sci. Technol..

[28] Rodriguez-Garcia G., Molinos-Senante M., Hospido A., Hernandez-Sancho F., Moreira M.T., Feijoo G. (2011). Environmental and economic profile of six typologies of wastewater treatment plants. Water Res..

[29] Zacharof M. (2017). Grape Winery Waste as Feedstock for Bioconversions: Applying the Biorefinery Concept. Waste Biomass Valor..

[30] Zacharof M.-P., Lovitt R.W. (2015). Adding value to wastewater by resource recovery and reformulations growth media: Current prospects and potential. J. Water Reuse Desalin..

[31] Bahri A., Jimenez B., Asano T. (2008). Case studies in Middle Eastern and North African countries. Water Reuse: An International Survey of Current Practice, Issues and Needs.

[32] Keraita B., Jimenez B., Drechsel P. (2008). Extent and implications of agricultural reuse of untreated, partly treated and diluted wastewater in developing countries. CAB Rev. Perspect. Agric. Vet. Sci. Nutr. Nat. Resour..

[33] Kelessidis A., Stasinakis A. (2012). Comparative study of the methods used for treatment and final disposal of sewage sludge in European countries. Waste Manag..

[34] Flores-Alsina X., Arnell M., Amerlinck Y., Corominas L., Gernaey K.V., Guo L., Lindblom E., Nopens I., Porro J., Shaw A. (2014). Balancing effluent quality, economic cost and greenhouse gas emissions during the evaluation of (plant-wide) control/operational strategies in WWTPs. Sci. Total Envrion..

[35] Flores-Alsina X., Corominas L., Snip L., Vanrolleghem P.A. (2011). Including greenhouse gas emissions during benchmarking of wastewater treatment plant control strategies. Water Res..

[36] Foladori P., Andreottola G., Ziglio G. (2010). Sludge Reduction Technologies in Wastewater Treatment Plants.

[37] Li W.-W., Yu H.-Q. (2011). From wastewater to bioenergy and biochemicals via two-stage bioconversion processes: A future paradigm. Biotechnol. Adv..

[38] Morrison M., Srinivasan R.S., Ries R. (2016). Complementary life cycle assessment of wastewater treatment plants: An integrated approach to comprehensive upstream and downstream impact assessments and its extension to building-level wastewater generation. Sustain. Cities Soc..

[39] Zhang X., Minear R.A. (2006). Removal of Low-Molecular Weight Dbps and Inorganic Ions for Characterization of High-Molecular Weight Dbps in Drinking Water. Water Res..

[40] Park N., Kwon B., Sun M., Ahn H., Kim C., Kwoak C., Lee D., Chae S., Hyung H., Cho J. (2005). Application Of Various Membranes To Remove Nom Typically Occurring In Korea With Respect To Dbp, Aoc And Transport Parameters. Desalination.

[41] Domany Z., Galambos I., Vatai G., Bekassy-Molnar E. (2002). Humic Substances Removal from Drinking Water by Membrane Filtration. Desalination.

[42] Al-Abri M., Al Anezi K., Dakheel A., Hilal N. (2010). Humic substance coagulation: Artificial neural network simulation. Desalination.

[43] Al-Abri M., Dakheel A., Tizaoui C., Hilal N. (2010). Combined humic substance and heavy metals coagula-tion, and membrane filtration under saline conditions. Desalination.

[44] Hilal N., Al-Abri M., Al-Hinai H., Somerfield C. (2008). Combined Humic Substance and Heavy Metals Agglomeration, and Membrane Filtration under Saline Conditions. Sep. Sci. Technol..

[45] Hakami M., Tizaoui C., Kochkodan V., Hilal N. (2012). Effect of Hydrodynamic Operations, Salinity, and Heavy Metals on HA Removal by Microfiltration Ceramic Tubular Membrane. Sep. Sci. Technol..

[46] Han Q., Lay H.T., Li W., Chew J.W. (2021). Effect of initial particle deposition rate on cake formation during dead-end microfiltration. J. Mem. Sci..

[47] Tchobanoglous G., Stensel H.D., Tsuchihashi R., Burton F.L., Abu-Orf M., Bowden G., Pfrang W. (2014). Wastewater Engineering: Treatment & Resource Recovery.

[48] Towler G.R., Sinnott R. (2013). Chemical Engineering Design Principles, Practice and Economics of Plant and Process Design.

[49] Yoshida H., Clavreul J., Scheutz C., Christensen T.H. (2014). Influence of data collection schemes on the Life Cycle Assessment of a municipal wastewater treatment plant. Water Res..

[50] Guo W., Ngo H.-H., Li J. (2012). A mini-review on membrane fouling. Bioresour. Technol..

[51] Van Der Bruggen B., Vandecasteele C. (2003). Removal of Pollutants from Surface Water and Groundwater by Nanofiltration: Overview of Possible Applications In The Drinking Water Industry. Environ. Pollut..

[52] Van Der Bruggen B., Verberk J.Q.J.C., Verhack J. (2004). Comparison of pressure-driven membrane processes and traditional processes for drinking water production in Europe based on specific impact criteria. Water SA.

[53] Baker R. (2000). Membrane Technology and Applications.

[54] Kaushik N. (2008). Membrane Separation Processes.

[55] (2011). World Demand for Membranes to Reach $19.3 Bil. https://www.flowcontrolnetwork.com/world-demand-for-membranes-to-reach-19-3-bil-in-2015/.

[56] J.E.T.O (2009). (JETRO) Rapid Growth of the Global Water Treatment Business-Japan’s Public and Private Sectors Join Hands to Develop National Strategy. https://www.jetro.go.jp/en/reports/market/pdf/2009_01.pdf.

[57] Scholz W., Lucas M. (2003). Techno-Economic Evaluation of Membrane Filtration for the Recovery and Re-Use of Tanning Chemicals. Water Res..

[58] Mo L., Huanga X. (2003). Fouling Characteristics and Cleaning Strategies in a Coagulation-Microfiltration Combination Process for Water Purification. Desalination.

[59] Chegini S. (2014). Removal of Potential Inhibitors from Hemicellulose Hydrolysate by Membrane Filtration. Master’s Thesis.

[60] Zacharof M., Lovitt R.W. (2013). Complex Effluent Streams as a Potential Source of Volatile Fatty Acids. Waste Biomass Valor..

[61] Kajenthira A., Siddiqi A., Anadon L.D. (2012). A new case for promoting wastewater reuse in Saudi Arabia: Bringing energy into the water equation. J. Environ. Manag..

[62] Kinčl J., Doleček P., Cakl J. (2009). Filtration Model for Hollow Fiber Membranes with Compressible Cake Formation. Desalination.

[63] Lee J., Ha J.-H., In-Hyuck Song I.-H., Shin D.W. (2017). Enhanced fouling resistance of organosilane-grafted ceramic microfiltration membranes for water treatment. J. Ceramic Soc. Jpn..

[64] Chang S., Fane A.G., Waite T.D. (2006). Analysis of Constant Permeate Flow Filtration Using Dead-End Hollow Fiber Membranes. J. Mem. Sci..

[65] Vyas H.K., Bennett R., Marshall A. (2000). Influence of Operating Conditions On Membrane Fouling In Crossflow Microfiltration Of Particulate Suspensions. Int. Dairy J..

[66] Teng C., Hawlader M., Malek A. (2003). An Experiment with Different Pretreatment Methods. Desalination.

[67] Tsakiris G., Alexakis D. (2013). Karstic Spring Water Quality: The Effect of Groundwater Abstraction from the Recharge Area. Desalin. Water Treat..

[68] Charcosset C. (2006). Membrane Processes in Biotechnology: An Overview. Biotechnol. Adv..

[69] Zacharof M.-P., Vouzelaud C., Lovitt R.W. (2014). The Use of Membrane Technology for the Formulation of Spent Anaerobic Digester Effluents as Nutrient Source for Bacterial Growth.

[70] Vinoth Kumara R., Goswamib L., Pakshirajanb K., GPugazhenthi G. (2016). Dairy wastewater treatment using a novel low cost tubular ceramic membrane and membrane fouling mechanism using pore blocking models. J. Water Process Eng..

[71] Zhang X., Linhua Fan L., Roddick F. (2018). Impact of the Interaction between Aquatic Humic Substances and Algal Organic Matter on the Fouling of a Ceramic Microfiltration Membrane. Membranes.

[72] Kumar C.M., Roshni M., Vasanth D. (2019). Treatment of aqueous bacterial solution using ceramic membrane prepared from cheaper clays: A detailed investigation of fouling and cleaning. J. Water Process Eng..

[73] Park W.-I., Jeong S., Im S.-J., Jang A. (2020). High turbidity water treatment by ceramic microfiltration membrane: Fouling identification and process optimization. Environ. Technol. Innov..

[74] Tang S., Zhang L., Peng Y., Liu J., Zhang Z. (2019). Fenton cleaning strategy for ceramic membrane fouling in wastewater treatment. J. Environ. Sci..

[75] Li W., Ling G.Q., Huang P., Li K., Lu H.Q., Hang F.X., Zhang Y., Xie C.F., Lu D.J., Li H. (2016). Performance of ceramic microfiltration membranes for treating carbonated and filtered remelt syrup in sugar refinery. J. Food Eng..

[76] Meng S., Zhang M., Yao M., Qiu Z., Hong Y., Lan W., Xia H., Jin X. (2019). Membrane Fouling and Performance of Flat Ceramic Membranes in the Application of Drinking Water Purification. Water.

[77] Sheikhi M., Arzani M., Mahdavi H.R., Mohammadi T. (2019). Kaolinitic clay-based ceramic microfiltration membrane for oily wastewater treatment: Assessment of coagulant addition. Ceram. Int..

[78] Jepsen K.L., Bram M.V., Pedersen S., Yang Z. (2018). Membrane Fouling for Produced Water Treatment: A Review Study from a Process Control Perspective. Water.

[79] Almojjly A., Johnson D., Mandale S., Hilal N. (2019). Optimisation of the removal of oil in water emulsion by using ceramic microfiltration membrane and hybrid coagulation/sand filter-MF. J. Water Process Eng..

[80] Anis S.F., Hashaikeh R., Hilal N. (2019). Microfiltration membrane processes: A review of research trends over the past decade. J. Water Process Eng..

[81] Tummons E., Qi H., Tanudjaja H.J., Hejase C.A., Chew J.W., Tarabara V.V. (2020). Membrane fouling by emulsified oil: A review. Sep. Purif. Technol..

[82] Nagasawa H., Omura T., Asai T., Kanezashi M., Tsuru T. (2020). Filtration of surfactant-stabilized oil-in-water emulsions with porous ceramic membranes: Effects of membrane pore size and surface charge on fouling behavior. J. Mem. Sci..

[83] Im D., Nakada N., Kato Y., Aoki M., Tanaka H. (2019). Pretreatment of ceramic membrane microfiltration in wastewater reuse: A comparison between ozonation and coagulation. Environ. Manag. J..

[84] Ebrahimi M., Willershausen D., Ashaghi K.S., Engel L., Placido P., Mund P., Bolduan P., Czermak P. (2010). Investigations on the use of different ceramic membranes for efficient oil-field produced water treatment. Desalination.

[85] Tanudjaja H.J., Hejase C.A., Tarabara V.V., Fane A.G., Chew J.W. (2019). Membrane-based separation for oily wastewater: A practical Perspective. Water Res..

[86] Garmsiri E., Rasouli Y., Abbasi M., Izadpanah A.A. (2017). Chemical cleaning of mullite ceramic microfiltration membranes which are fouled during oily wastewater treatment. J. Water Process Eng..

[87] Yaser Rasouli Y., Abbasi M., Hashemifard S.A. (2019). Fabrication, characterization, fouling behavior and performance study of ceramic microfiltration membranes for oily wastewater treatment. Asian Ceram. Soc. J..

[88] Li M., Zhao Y., Zhou S., Xing W. (2010). Clarification of raw rice wine by ceramic microfiltration membranes and membrane fouling analysis. Desalination.

[89] Hankins N.P., Lu N., Hilal N. (2006). Enhanced Removal of Heavy Metal Ions Bound To Humic Acid by Polyelectrolyte Flocculation. Sep. Purif. Technol..

[90] Hsu B.-M., Yeh H.-H. (2003). Removal of Giardia and Cryptosporidium in Drinking Water Treatment: A Pilot-Scale Study. Water Res..

[91] Bottino A., Capannelli C., Del Borghi A., Colombino MConio O. (2001). Water Treatment for Drinking Purpose: Ceramic Microfiltration Application. Desalination.

[92] Mulder M. (1991). Basic Principles on Membrane Technology.

[93] Mulder M. (1994). The Use of Membrane Processes in Environmental Problems. An Introduction. Nato Asi Ser. E Appl. Sci. Adv. Study Inst..

[94] Nah W., Kang Y.W., Hwang K.-Y., Song W.-K. (2000). Mechanical pre-treatment of waste activated sludge for anaerobic digestion process. Water Res..

[95] Araki K., Sakai H. (2011). Ceramic membrane development in NGK. IOP Conference Series: Materials Science Engineering.

[96] Parameshwaran K., Fane A.G., Cho B.D., Kim K.J. (2001). Analysis of microfiltration performance with constant flux processing of secondary effluent. Water Res..

[97] Ciora R.J., Liu P.K.T. (2003). Ceramic membranes for enviromental related applications, Fluid Part. Sep. J..

[98] Kang I.-J., Yoon S.-H., Lee C.-H. (2002). Comparison of the filtration characteristics of organic and inorganic membranes in a membrane-coupled anaerobic bioreactor. Water Res..

[99] Waeger F., Delhaye T., Fuchs W. (2010). The use of ceramic microfiltration and ultrafiltration membranes for particle removal from anaerobic digester effluents. Sep. Purif. Technol..

[100] Kim J.-O., Kim S.-K., Kim R.-H. (2010). Filtration performance of ceramic membrane for the recovery of volatile fatty acids from liquid organic sludge. Desalination.

[101] Mosqueda-Jimenez D.B., Huck P.M. (2006). Characterization of Membrane Foulants in Drinking Water Treatment. Desalination.

[102] Xu N., Xing W., Xu N., Shi J. (2003). Study on Ceramic Membrane Bioreactor with Turbulence Promoter. Sep. Purif. Technol..

[103] Sotiropoulou S., Gavalas V., Vamvakaki V., Chaniotakis N. (2003). Novel Carbon Materials in Biosensor Systems. Biosens. Bioelectr..

[104] Yoshikawa M., Yonetani K. (2002). Molecularly Imprinted Polymeric Membranes with Oligopeptide Tweezers for Optical Resolution. Desalination.

[105] Han S.K., Na K., Bae Y.H. (2003). Sulfonamide Based Ph-Sensitive Polymeric Micelles: Physicochemical Characteristics and pH-Dependent Aggregation. Colloids Surf. A Physicochem. Eng. Asp..

[106] Judd S., Jefferson B. (2003). Membranes for Industrial Wastewater Recovery and Re-Use.

[107] Judd S.J., Hillis P. (2001). Optimisation of Combined Coagulation and Microfiltration for Water Treatment. Water Res..

[108] Hua F., Tsang Y., Wang Y., Chan S., Chua H., Sin S. (2007). Performance Study of Ceramic Microfiltration Membrane For Oily Wastewater Treatment. Chem. Eng. J..

[109] Xu N., Xing W., Xu N., Shi J. (2002). Application of Turbulence Promoters in Ceramic Membrane Bioreactor Used for Municipal Wastewater Reclamation. Membr. Sci. J..

[110] Krstić D.M., Koris A.K., Tekić M.N. (2006). Do Static Turbulence Promoters Have Potential in Crossflow Membrane Filtration Applications?. Desalination.

[111] Brinker C.J., Scherer G.W. (1990). Sol-Gel Science: The Physics and Chemistry of Sol-Gel Processing.

[112] Li F., Yang Y., Fan Y., Xing W., Wang Y. (2012). Modification of Ceramic Membranes for Pore Structure Tailoring: The Atomic Layer Deposition Route. Membr. Sci. J..

[113] Li M., Wu G., Guan Y., Zhang X. (2011). Treatment of river water by a hybrid coagulation and ceramic membrane process. Desalination.

[114] Li W., Zhou L., Xing W., Xu N. (2010). Coagulation-microfiltration for lake water purification using ceramic membranes. Desalin. Water Treat..

[115] Agashichev S.P. (2006). Enhancement of Concentration Polarization Due to Gel Accumulated At Membrane Surface. Membr. Sci. J..

[116] Bacchin P., Si-Hassen D., Starov V., Clifton M.J., Aimar P. (2002). A Unifying Model for Concentration Polarization, Gel-Layer Formation and Particle Deposition in Cross-Flow Membrane Filtration of Colloidal Suspensions. Chem. Eng. Sci..

[117] Zhong Z., Xing W., Zhang B. (2013). Fabrication of Ceramic Membranes with Controllable Surface Roughness and Their Applications in Oil/Water Separation. Ceram. Int..

[118] Meyn T., Leiknes T. (2010). Comparison of optional process configurations and operating conditions for ceramic membrane MF coupled with coagulation/flocculation pre-treatment for the removal of NOM in drinking water production. J. Water Supply Res. Technol..

[119] Xing W., Fan Y., Zhong Z., Xu N. (2009). Recent Advances in Process-Engineering Oriented Preparation and Application of Ceramic Membranes. J. Chem. Ind. Eng. Soc. China.

[120] Del Colle R., Fortulan C.A., Fontes S.R. (2011). Manufacture and Characterization of Ultra and Microfiltration Ceramic Membranes by Isostatic Pressing. Ceramics Int..

[121] Lim A.L., Bai R. (2003). Membrane Fouling and Cleaning In Microfiltration of Activated Sludge Wastewater. J. Membr. Sci..

[122] Ma H., Bowman C.N., Davis R.H. (2000). Membrane Fouling Reduction by Backpulsing and Surface Modification. Membr. Sci. J..

[123] Ma H.M., Hakim L.F., Bowman C.N., Davis R.H. (2001). Factors Affecting Membrane Fouling Reduction by Surface Modification and Backpulsing. Membr. Sci. J..

[124] Ma J., Liu W. (2002). Effectiveness of Ferrate (Vi) Preoxidation in Enhancing the Coagulation of Surface Waters. Water Res..

[125] Al-Ahmad M., Aleem F.A.A., Mutiri A., Ubaisy A. (2000). Biofuoling in RO Membrane Systems Part 1: Fundamentals and Control. Desalination.

[126] Ahmad A.L., Mariadas A. (2004). Baffled Microfiltration Membrane and Its Fouling Control for Feed Water of Desalination. Desalination.

[127] Taniguchi M., Kilduff J.E., Belfort G. (2003). Low Fouling Synthetic Membranes by UV-Assisted Graft Polymerization: Monomer Selection to Mitigate Fouling by Natural Organic Matter. Membr. Sci. J..

[128] Bian R., Yamamoto K., Watanabe Y. (2000). The Effect of Shear Rate on Controlling the Concentration Polarization and Membrane Fouling. Desalination.

[129] Li H., Wu S., Du C., Zhong Y., Yang C. (2020). Preparation, Performances, and Mechanisms of Microbial Flocculants for Wastewater Treatment. Int. J. Environ. Res. Public Health.

[130] Yiantsios S.G., Karabelas A.J. (2001). An Experimental Study of Humid Acid and Powdered Activated Carbon Deposition on UF Membranes and Their Removal by Backwashing. Desalination.

[131] Katsoufidou K., Yiantsios S., Karabelas A. (2008). An Experimental Study of UF Membrane Fouling By Humic Acid and Sodium Alginate Solutions: The Effect of Backwashing on Flux Recovery. Desalination.

[132] Schäfer A.I., Fane A.G., Waite T.D. (2000). Fouling Effects on Rejection in the Membrane Filtration of Natural Waters. Desalination.

[133] Schäfer A.I., Fane A.G., Waite T.D. (2001). Cost Factors and Chemical Pretreatment Effects in the Membrane Filtration of Waters Containing Natural Organic Matter. Water Res..

[134] Schäfer A.I., Richards B.S. (2005). Testing of a Hybrid Membrane System for Groundwater Desalination in an Australian National Park. Desalination.

[135] Schäfer A.I., Schwicker U., Fischer M.M., Fane A.G., Waite T.D. (2000). Microfiltration of Colloids and Natural Organic Matter. J. Membr. Sci..

[136] Hassan A.M., Farooque A.M., Jamaluddin A.T.M., Al-Amoudi A.S., Al-Sofi M.A.K., Al-Rubaian A.F., Kither N.M., Al-Tisan I.A.R., Rowaili A. (2000). A Demonstration Plant Based on The New NF—SWRO Process. Desalination.

[137] Brehant A., Bonnelye V., Perez M. (2002). Comparison of MF/UF Pretreatment with Conventional Filtration Prior To Ro Membranes for Surface Seawater Desalination. Desalination.

[138] Van Reis R., Zydney A. (2007). Bioprocess membrane technology. Membr. Sci. J..

[139] Jones K.L., O’Melia C.R. (2000). Protein and Humic Acid Adsorption onto Hydrophilic Membrane Surfaces: Effects of pH And Ionic Strength. Membr. Sci. J..

[140] Lauterböck B., Nikolausz M., Lv Z., Baumgartner M., Liebhard G., Fuchs W. (2014). Improvement of anaerobic digestion performance by continuous nitrogen removal with a membrane contactor treating a substrate rich in ammonia and sulfide. Bioresour. Technol..

[141] Zularisam A., Ismail A., Sakinah M. (2010). Application and Challenges of Membrane in Surface Water Treatment. Appl. Sci. J..

[142] Pan Y., Li H., Zhang X., Li A. (2016). Characterization of natural organic matter in drinking water: Sample preparation and analytical approaches. Trends Environ. Anal. Chem..

[143] Bratby J. (2006). Coagulation and Flocculation in Water and Wastewater Treatment.

[144] Duan J., Gregory J. (2003). Coagulation by Hydrolyzing Metal Salts. Adv. Colloid Interface Sci..

[145] Gregory J. (2006). Particles in Water: Properties and Processes.

[146] Gregory J., Yukselen M., Hahn H., Hoffmann E., Odegaard H. (2002). Break-Up and Re-Formation of Flocs Formed by Hydrolyzing Coagulants And Polymeric Flocculants. Chemical Water and Wastewater Treatment Vii.

[147] Al-Mutairi N.Z., Hamoda M.F., Al-Ghusain I. (2004). Coagulant Selection and Sludge Conditioning in A Slaughterhouse Wastewater Treatment Plant. Biores. Technol..

[148] Kam S.-K., Gregory J. (2001). The Interaction of Humic Substances with Cationic Polyelectrolytes. Water Res..

[149] Amy G. (2008). Fundamental Understanding of Organic Matter Fouling of Membranes. Desalination.

[150] Nozaic D., Freese S., Thompson P. (2001). Longterm Experience in the Use of Polymeric Coagulants at Umgeni Water. Water Sci. Technol. Water Supply.

[151] Gabelich C.J., Yun T.I., Coffey B.M., Suffet I.H.M. (2002). Effects of Aluminum Sulfate and Ferric Chloride Coagulant Residuals on Polyamide Membrane Performance. Desalination.

[152] Bolto B., Gregory J. (2007). Organic Polyelectrolytes in Water Treatment. Water Res..

[153] Bolto B.A., Gayle N., David D. (2006). Chapter 5: Coagulation and Flocculation with Organic Polyelectrolytes. Interface Science and Technology.

[154] Prime D.C., Stapley A.G., Rielly C.D., Jones J.R., Leaper M.C. (2011). Analysis of Powder Caking In Multicomponent Powders Using Atomic Force Microscopy To Examine Particle Properties. Chem. Eng. Technol..

[155] Konieczny K., Bodzek M., Rajca M.A. (2006). Coagulation–MF System for Water Treatment Using Ceramic Membranes. Desalination.

[156] Konieczny K., Rajca M., Bodzek M., Kwiecińska A. (2009). Water Treatment Using Hybrid Method of Coagulation and Low-Pressure Membrane Filtration. Environ. Prot. Eng..

[157] Nishi L., Vieira A.M.S., Vieira M.F., Silva G.F., Bergamasco R. (2012). Application of Hybrid Process of Coagulation/Flocculation and Membrane Filtration for The Removal of Protozoan Parasites from Water. Procedia Eng..

[158] Chesters S., Darton E., Gallego S., Vigo F. (2009). The Safe Use of Cationic Flocculants with Reverse Osmosis Membranes. Desalin. Water Treat..

[159] Farahbakhsh K., Svrcek C., Guest R., Smith D.W. (2004). A Review of the Impact of Chemical Pretreatment on Low-Pressure Water Treatment Membranes. Environ. Eng. Sci. J..

[160] Katayon S., Noor M.M.M., Ghani L.A., Ahmad J. (2005). Influence of Cationic Polyelectrolyte Coagulant on Microfiltration Performance for Treatment of Oxidation Pond Effluent. Desalination.

[161] Katayon S., Noor M.M.M., Tat W.K., Halim G.A., Thamer A.M., Badronisa Y. (2007). Effect of Natural Coagulant Application on Microfiltration Performance in Treatment of Secondary Oxidation Pond Effluent. Desalination.

[162] Koseoglu H., Yigit N.O., Iversen V., Drews A., Kitis M., Lesjean B., Kraume M. (2008). Effects of Several Different Flux Enhancing Chemicals on Filterability and Fouling Reduction of Membrane Bioreactor (MBR) Mixed Liquors. Membr. Sci. J..

[163] Carroll T., King S., Gray S.R., Bolto B.A., Booker N.A. (2000). The Fouling of Microfiltration Membranes by NOM after Coagulation Treatment. Water Res..

[164] Abdessemed D., Nezzal G. (2003). Treatment of Primary Effluent by Coagulation-Adsorption-Ultrafiltration for Reuse. Desalination.

[165] Gaydardzhiev S., Karthikeyan J., Ay P. (2006). Colour Removal from Model Solutions by Coagulation-Surface Charge and Floc Characterisation Aspects. Environ. Technol..

[166] Jarvis P., Jefferson B., Gregory J., Parsons S.A. (2005). A Review of Floc Strength and Breakage. Water Res..

[167] Yu W., Liu T., Crawshaw J., Liu T., Graham N. (2018). Ultrafiltration and nanofiltration membrane fouling by natural organic matter: Mechanisms and mitigation by pre-ozonation and pH. Water Res..

[168] Moulin P., Veyret D., Charbit F. (2001). Dean Vortices: Comparison of Numerical Simulation of Shear Stress and Improvement of Mass Transfer in Membrane Processes at Low Permeation Fluxes. Membr. Sci. J..

[169] Moll R., Moulin P., Veyret D., Charbit F. (2002). Numerical Simulation of Dean Vortices: Fluid Trajectories. Membr. Sci. J..

[170] Ghidossi R., Veyret D., Moulin P. (2006). Computational Fluid Dynamics Applied to Membranes: State of The Art and Opportunities. Chem. Eng. Process..

[171] Thomas H., Judd S., Murrer J. (2000). Fouling Characteristics of Membrane Filtration in Membrane Bioreactors. Membr. Technol..

[172] Nandi B.K., Moparthi A., Uppaluri R., Purkait M.K. (2010). Treatment of oily wastewater using low cost ceramic membrane: Comparative assessment of pore blocking and artificial neural network models. Chem. Eng. Res. Des..

[173] Wakeman R.J., Williams C.J. (2002). Additional Techniques to Improve Microfiltration. Sep. Purif. Technol..

[174] Pervov A.G., Andrianov A.P., Efremov R.V., Desyatov A.V., Baranov A.E. (2003). A New Solution for the Caspian Sea Desalination: Low-Pressure Membranes. Desalination.

[175] Porcelli N., Judd S. (2010). Chemical Cleaning of Potable Water Membranes: A Review. Sep. Purif. Technol..

[176] Saffaj N., MPersin M., Younsi S.A., Albizane M., Cretin A. (2006). Larbot, Elaboration and characterization of microfiltration and ultrafiltration membranes deposited on raw support prepared from natural moroccan clay: Application to filtration of solution containing dyes and salts. Appl. Clay Sci..

[177] Vasanth D., Pugazhenthi G., Uppaluri R. (2011). Fabrication and properties of low-cost ceramic microfiltration membranes for separation of oil and bacteria from its solution. J. Membr. Sci..

[178] He Z., Lyu Z., Gu Q., Zhang L., Wang J. (2019). Ceramic-based membranes for water and wastewater treatment. Colloids Surf. A Physicochem. Eng. Asp..

[179] Lee N., Amy G., Croué J.-P., Buisson H. (2004). Identification and Understanding Of Fouling In Low-Pressure Membrane (MF/UF) Filtration By Natural Organic Matter (NOM). Water Res..

[180] Lee S., Kwon B., Sun M., Cho J. (2005). Characterizations of Nom Included in NF and UF Membrane Permeates. Desalination.

[181] Al-Amoudi A., Lovitt R.W. (2007). Fouling Strategies and the Cleaning System of NF Membranes and Factors Affecting Cleaning Efficiency. J. Membr. Sci..

[182] Kumar R.V., Ghoshal A.K., Pugazhenthi G. (2015). Elaboration of novel tubular ceramic membrane from inexpensive raw materials by extrusion method and its performance in microfiltration of synthetic oily wastewater treatment. J. Membr. Sci..

[183] Bird M.R., Bartlett M. (2002). Measuring and Modelling Flux Recovery During The Chemical Cleaning Of MF Membranes For The Processing Of Whey Protein Concentrate. J. Food Eng..

[184] Strugholtz S., Sundaramoorthy K., Panglisch S., Lerch A., Brugger A., Gimbel R. (2005). Evaluation of the Performance of Different Chemicals For Cleaning Capillary Membranes. Desalination.

[185] Zondervan E., Roffel B. (2007). Evaluation of Different Cleaning Agents Used For Cleaning Ultra Filtration Membranes Fouled By Surface Water. J. Membr. Sci..

[186] Hofs J., Ogier D., Vries E.F., Beerendonk E.R. (2011). Cornelissen, Comparison of ceramic and polymeric membrane permeability and fouling using surface water. Sep. Purif. Technol..

[187] Mao H., Bu J., Qiu M., Ding D., Chen X., Verweij H., Fan Y. (2019). PZT/Ti composite piezoceramic membranes for liquid filtration: Fabrication and self-cleaning properties. J. Membr. Sci..

[188] Alresheedi M.T., Barbeau B., Basu O.D. (2019). Comparisons of NOM fouling and cleaning of ceramic and polymeric membranes during water treatment. Sep. Purif. Technol..

[189] Chen H., Kim A.S. (2006). Prediction of Permeate Flux Decline in Crossflow Membrane Filtration of Colloidal Suspension: A Radial Basis Function Neural Network Approach. Desalination.

[190] Cheng W.P. (2002). Comparison of Hydrolysis/Coagulation Behavior of Polymeric and Monomeric Iron Coagulants in Humic Acid Solution. Chemosphere.

[191] Cheryan M. (1998). Fouling and Cleaning: Cleaning Membranes. Ultrafiltration and Microfiltration Handbook.

[192] Cheryan M. (2000). Ultrafiltration and Microfiltration Handbook.

[193] Hristov A., Djambazov I., Dimitrov D. (2012). Preparation and characterization of porous ceramic membranes for micro-filtration from natural zeolite. J. Chem. Technol. Metall..

[194] Côté P., Siverns S., Monti S. (2005). Comparison of Membrane-Based Solutions for Water Reclamation and Desalination. Desalination.

[195] Curcio S., Calabrò V., Iorio G. (2006). Reduction and Control of Flux Decline in Cross-Flow Membrane Processes Modeled By Artificial Neural Networks. J. Membr. Sci..

[196] Di Bella G., Di Trapani D. (2019). A Brief Review on the Resistance-in-Series Model in Membrane Bioreactors (MBRs). Membranes.

[197] De La Rubia A., Rodríguez M., León V.M., Prats D. (2008). Removal of Natural Organic Matter and Thm Formation Potential by Ultra-and Nanofiltration of Surface Water. Water Res..

[198] Gu Q., Ng T.C.A., Zhang L., Lyu Z., Zhang Z., Ng H.Y., Wang J. (2020). Interfacial diffusion assisted chemical deposition (ID-CD) for confined surface modification of alumina microfiltration membranes toward high flux and anti-fouling. Sep. Purif. Technol..

[199] Sajjadnejad M., Haghshenas S.M.S., Tavakoli Targhi V., Ghafarian Zahmatkesh H., Naeimi M. (2020). Utilization of Sustainable Energies for Purification of Water. Adv. J. Chem. A.

[200] El Tabach E., Lancelot L., Shahrour I., Najjar Y. (2007). Use of Artificial Neural Network Simulation Metamodelling To Assess Groundwater Contamination in A Road Project. Math. Comput. Model..

[201] He C., Vidic R.D. (2016). Application of microfiltration for the treatment of Marcellus Shale flow backwater: Influence of floc breakage on membrane fouling. J. Membr. Sci..

[202] Jiang Q., Rentschler J., Perrone R., Liu K. (2013). Application of ceramic membrane and ion-exchange for the treatment of the flowback water from Marcellus Shale gas production. J. Membr. Sci..

[203] Li M., Zhao Y., Zhou S., Xing W., Wong F.S. (2007). Resistance analysis for ceramic membrane microfiltration of raw soy sauce. J. Membr. Sci..

[204] Zhang X., Fan L., Roddick F.A. (2014). Feed water coagulation to mitigate the fouling of a ceramic MF membrane caused by soluble algal organic matter. Sep. Purif. Technol..

[205] Klavins M., Ansone L. (2010). Study of Interaction between humic Acids and Fullerene C 60 Using Fluorescence Quenching Approach. Ecol. Chem. Eng..

[206] Kloster N., Brigante M., Zanini G., Avena M. (2013). Aggregation Kinetics of Humic Acids in The Presence Of Calcium Ions. Colloids Surf. A Physicochem. Eng. Asp..

[207] Chang Q., Zhou J.E., Wang Y., Liang J., Zhang X., Cerneaux S., Wang X., Zhu Z., Dong Y. (2014). Application of ceramic microfiltration membrane modified by nano-TiO2 coating in separation of a stable oil-in-water emulsion. J. Membr. Sci..

[208] Chang Q., Zhou J.E., Wang Y., Wang J., Meng G. (2010). Hydrophilic modification of Al_2_O_3_ microfiltration membrane with nano-sized γ-Al_2_O_3_ coating. Desalination.

[209] Machenbach I. (2007). Drinking Water Production by Coagulation and Membrane Filtration. Ph.D. Thesis.

[210] Zhao Y., DLu D., Cao Y.S., Luo S., Zhao Q., Yang M., CXu C., Ma J. (2018). Interaction analysis between gravity-driven ceramic membrane and smaller organic matter: Implications for retention and fouling mechanism in ultralow pressure-driven filtration system. Environ. Sci. Technol..

[211] Low S.C., Han H.J., Jin W.X. (2004). Characteristics of a Vibration Membrane in Water Recovery from Fine Carbon-Loaded Wastewater. Desalination.

[212] Ogunbiyi O.O., Miles N.J., Hilal N. (2007). Comparison of Different Pitch Lengths on Static Promoters for Flux Enhancement in Tubular Ceramic Membrane. Sep. Purif. Technol..

[213] Al-Shammari S.B., Bou-Hamad S., Al-Saffar A., Salman M., Al-Sairafi A. (2015). Treatment of dairy processing wastewater using integrated submergedmembrane microfiltration system. J. Environ. Anal. Toxicol..

[214] Palmer S.J. (2010). Future Challenges to Asset Investment in the UK Water Industry: The Wastewater Asset Investment Risk Mitigation Offered By Minimizing Principal Operating Cost Risks. Water Clim. Chang..

[215] Papić S., Koprivanac N., Lončarić Božić A., Meteš A. (2004). Removal of Some Reactive Dyes from Synthetic Wastewater By Combined Al(Iii) Coagulation/Carbon Adsorption Process. Dye Pigment..

[216] Prisciandaro M., Salladini A., Barba D. (2008). Membrane Filtration of Surface Water for the Removal of humic Substances. Chem. Eng. Trans..

[217] Qdais H.A., Moussa H. (2004). Removal of Heavy Metals from Wastewater by Membrane Processes: A Comparative Study. Desalination.

[218] Ladner D.A., Vardon D.R., Clark M.M. (2010). Effects of shear on microfiltration and ultrafiltration fouling by marine bloom-formingalgae. J. Membr. Sci..

[219] Lee S.-J., Dilaver M., Park P.-K., Kim J.-H. (2013). Comparative analysis of fouling characteristics of ceramic and polymeric microfiltration membranes using filtration models. J. Membr. Sci..

[220] Park S., Kang J.S., Lee J.J., Vo T.K.Q., Kim H.S. (2018). Application of physical and chemical enhanced backwashing to reduce membrane fouling in the water treatment process using ceramic membranes. Membranes.

[221] Lohaus J., Stockmeier F., Surray P., Lölsberg J., Wessling M. (2020). What are the microscopic events during membrane backwashing?. J. Membr. Sci..

